# The Replication of *Frataxin* Gene Is Assured by Activation of Dormant Origins in the Presence of a GAA-Repeat Expansion

**DOI:** 10.1371/journal.pgen.1006201

**Published:** 2016-07-22

**Authors:** Martina Stevanoni, Elisa Palumbo, Antonella Russo

**Affiliations:** Department of Biology, University of Padova, Padova, Italy; The Hospital for Sick Children and University of Toronto, CANADA

## Abstract

It is well known that DNA replication affects the stability of several trinucleotide repeats, but whether replication profiles of human loci carrying an expanded repeat differ from those of normal alleles is poorly understood in the endogenous context. We investigated this issue using cell lines from Friedreich’s ataxia patients, homozygous for a GAA-repeat expansion in intron 1 of the *Frataxin* gene. By interphase, FISH we found that in comparison to the normal *Frataxin* sequence the replication of expanded alleles is slowed or delayed. According to molecular combing, origins never fired within the normal *Frataxin* allele. In contrast, in mutant alleles dormant origins are recruited within the gene, causing a switch of the prevalent fork direction through the expanded repeat. Furthermore, a global modification of the replication profile, involving origin choice and a differential distribution of unidirectional forks, was observed in the surrounding 850 kb region. These data provide a wide-view of the interplay of events occurring during replication of genes carrying an expanded repeat.

## Author Summary

The expansion of trinucleotide repeats (TNR) is associated with a large number of human neurodegenerative and neuromuscular diseases, among which the most known are Friedreich's ataxia (GAA/TTC), Huntington's disease (CAG/CTG), and myotonic dystrophy (CTG/CAG). TNR are among the most unstable DNA regions in the genome, and an important step in their expansion is the attainment of a threshold-length. This process occurs during paternal or maternal gametogenesis, leading to an earlier onset of the disease in the next generation. The severity of the disease is strictly related to the TNR length. The repeat instability results from non-B DNA secondary structures formed during DNA replication, repair, recombination and gene transcription. However, the pathways leading to expansion remain poorly understood. Here, we describe the effects of the GAA-expansion on the DNA replication of *Frataxin* gene. By analyzing the replication profile of mutated and normal genotypes, we have found that the replication of the expanded gene is slowed or delayed in comparison with the non-expanded condition. Interestingly, this observation accords to a global modification of the replication profile affecting the usage of both replication forks and origins, which can be referred as the functional units of any replication program.

## Introduction

During DNA replication the cell must be ready to face diverse potential obstacles to fork progression, including changes in chromatin organization, variations in cellular environment, formation of secondary structures [1–3]. To deal with these adverse conditions and ensure accurate genome duplication, mammalian cells rely on the plasticity of the replication process, which can be appreciated both at the global and local level [4–6].

It is well-known that DNA replication may affect the stability of several trinucleotide repeats [7–9]. Evidence was accumulated by a wide range of experimental systems, including bacteria, yeast, transfected or engineered human cells [8,10–13]. However, whether replication profiles of human loci carrying an expanded repeat differ from those of normal alleles is poorly understood in the endogenous context. A fine characterization of the replication profiles of loci involved in trinucleotide-expansion human diseases could be of general interest, because knowledge concerning the replication dynamics at unstable genomic regions is still limited [4,14]. In addition, this information could help to define the replication-based mechanisms causing instability of trinucleotide repeats [7,15].

In relation to the orientation of the repeat and the distance from a replication origin, secondary structures may have a diverse potential to be formed, to be stable, and eventually to cause replication impediments and trinucleotide length variations [16]. One model called origin-switch predicts that a change in the position of a replication origin across the repeat may lead to opposite orientations of normal and expanded alleles in the two template strands [17–19]. A recent study describing the replication profile of the *FMR1* locus, which is involved in fragile X syndrome when a CGG-repeat is expanded, strongly support an origin-switch mechanism at the basis of the CGG-repeat expansion in early developmental stages [20].

Human subjects affected by Friedreich’s ataxia (FRDA) are homozygous for a GAA-repeat expansion in intron 1 of *Frataxin* (*FXN*) gene [21], a mutation causing the transcriptional inhibition of the gene [22–24]. In proximity of the expansion the chromatin is remodeled leading to sequence heterochromatinization [25–28]. Somatic instability of the expansion has been reported in several tissues of FRDA patients and the same effect may be observed in mutated lymphoblastoid cell lines [29–31]. There is large agreement concerning the involvement of a replication-based mechanism at the origin of GAA-repeat instability in FRDA patients. However, evidence was mainly derived from model systems, and by tracking the events occurring at the repeated sequence only [10–12,32].

To verify if mammalian cells modulate origin usage and fork rates in the presence of long stretches of GAA repeats, we used cell lines derived from FRDA patients. A mild shift of *FXN* replication timing was detected in patients’ cells. By monitoring fork progression in a wide genomic segment surrounding *FXN*, we found evidence for recruitment of dormant origins, which may be consistent with an origin-switch effect at the GAA-expanded repeat.

## Results

### Cell line characterization

GM15850 and GM16227 cell lines (from two unrelated FRDA patients), and GM15851 cells derived from the healthy brother of patient GM15850, were obtained from the Coriell cell line repository. In the lack of available cell samples from healthy relatives of the second patient, the EBV-immortalized B lymphoblastoid H691 cell line, derived from a male subject and previously used in our laboratory for replication studies [14], was used as a further control.

The three Coriell cell lines were thoroughly characterized by genotype, transcriptional, cell cycle and replication analysis (S1 Fig). *FXN* genotype and transcriptional activity were assessed also in the H691 cell line (S1B and S1C Fig).

The size of the GAA-repeat expansion in the two patients’ cell lines was evaluated by long-range PCR at the beginning and the conclusion of the study. The results were in agreement with the information provided by the Coriell cell repository; furthermore, somatic instability of the expansion was excluded on the basis of the lack of multiple bands relative to the amplification of the expanded GAA-repeats (S1A and S1B Fig).

As expected, the transcriptional inhibition of the mutated alleles was observed in the patients’ cell lines (S1C Fig).

According to flow cytometry-based cell cycle distributions (S1D Fig), no detectable differences were found between mutated and normal cells. In addition, when DNA replication profiles were assayed by molecular combing (S1E Fig), both replication fork rates and Inter-Origin Distances (IOD) fell into the ranges known for lymphoblastoid cells [14,33].

### Replication timing of the *FXN* gene

**Fig 1:**
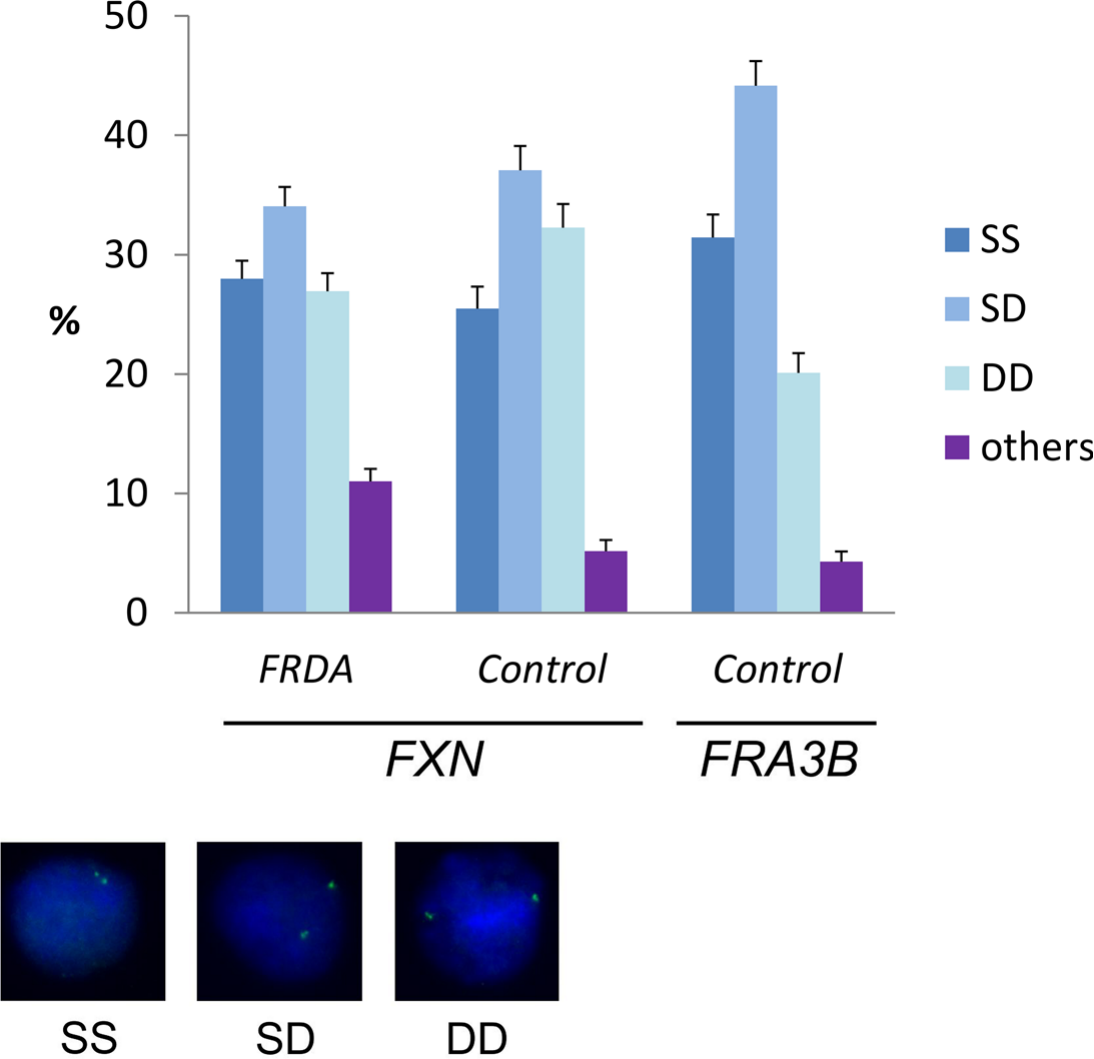
Replication timing of mutant and normal *FXN* alleles. Percentages are calculated from pooled data obtained with *FRDA* (GM15850 and GM16227) and control GM15851 cells. Per each group, at least 550 nuclei were analyzed from at least two independent replicated experiments (Raw data in S1 Table). SS = nuclei with two single FISH spots (non-replicated alleles); SD = nuclei with one single and one duplicated FISH signal (one allele has been replicated); DD = nuclei with two duplicated FISH signals (both alleles have been replicated); others = nuclei with one or none FISH signals. Error bars indicate standard errors of proportions. The probe used in these experiments is BAC RP11-265B8. For comparison, the replication timing of a late replication sequence (*FRA3B*, probe RP11-468L11) in normal GM15851 cells is shown. Examples of FISH replication patterns are shown in the bottom of the Figure.

According to data provided by the Encode project *FXN* is harbored in a mid-late replicating domain [34]. We wondered if the long GAA-repeat expansion found in mutant alleles (ranging 630–1030 repeats in our samples) could affect the replication timing of the gene. To answer this question, interphase FISH experiments were performed in FRDA cells (GM15850 and GM16227 cell lines) and in control cells GM15851, under the assumption that nuclei showing two single FISH spots (SS) carried non-replicated alleles, while cells showing a pair of duplicated FISH signals (DD) had already completed the replication of both alleles. Asynchronous patterns with a single and a duplicated FISH signal (SD) can also be observed [35]. In the case of *FXN* gene, these patterns were similarly represented in the three cell populations, independent of the presence of a mutated or a normal pair of alleles (Fig 1; S1 Table). This result might indicate that no differences exist between mutant and normal *FXN* alleles. In parallel, the late replicating sequence of the common fragile site *FRA3B* was evaluated as a positive control. This locus displayed a DD pattern in less than 25% nuclei (Fig 1; S1 Table), suggesting that *FRA3B* replication is slightly postponed with respect to the *FXN* timing. From this data we could confirm a mid-late replication timing for *FXN* locus.

However, we reasoned that the sensitivity of the interphase approach could not be sufficient to detect mild temporal replication shifts, in particular if mid-late or late replicating regions are investigated. Indeed, in mid-late replicating domains the activation of origins is less efficient and more stochastic than in early domains, leading to an increase of cell-to-cell variability [36,37]. Thus, to examine in depth the replication timing of *FXN* we performed FACS sorting experiments coupled with interphase FISH. Four cell fractions of identical size and corresponding to early-to-late S-phase (S1-S4) were isolated, and the level of contamination recorded by post-sorting FACS analysis appeared very small (S2 Fig).

As sorted cells have been CldU-labeled immediately before harvesting, in the course of the following microscope analyses nuclei could be classified as early, mid or late S-phase by CldU-immunodetection, giving a second control of the accuracy of cell separation (S3 Fig, S2 Table). Coherently with the post-sorting FACS analyses (S2 Fig), in the 4 fractions the large majority of the cells were positive to CldU labeling and belonged to the expected substage of the S-phase (early-mid in S1-S2 fractions, mid-late in S3-S4 fractions).

Interphase FISH data concerning the normal cell line GM15851 were obtained in two independent sorting experiments and were remarkably reproducible (see S2 Table for raw data). Table 1 gives the summary of these analyses, further supporting the indication of a mid-late replication timing for the wildtype *FXN* locus: indeed, according to the percentage of DD nuclei observed in S2, within the first half of the S-phase only 25% of GM15851 cells have completed the replication of this sequence (Table 1). The replication patterns observed in the S1 and S2 fractions isolated from normal and mutant cells were significantly different when compared by chi-square analysis (P < 0.05 for early S-phase cells; P < 0.001 for mid S-phase cells). The observed trends indicate a faster replication progression of the wildtype than the mutant *FXN* allele, demonstrated in particular by the excess of SS nuclei persisting in mutant S2 cells (Table 1, S2 Table). In the S3 fraction a significant variation was recorded between replication patterns of late S-phase mutant and normal cells (P < 0.05); in the S4 fraction a statistical difference was detected when comparing the replication patterns of mid S-phase mutant with that of the normal cells (P < 0.001). These observations can be interpreted as a downstream effect of the earlier shift of the replication timing, as the proportions of SS nuclei were not involved in these variations (Table 1). In fact, in the second half of the S-phase both normal and expanded *FXN* alleles are undergoing and completing their replication.

**Table 1:** Replication timing of normal and mutant *FXN* alleles according to interphase FISH after FACS cell sorting.

Cell sample	Cell fraction	Replication patterns^#^	Total cells	S-phase cells^#^ N	early S-phase^#^ N (% ± SE)^§^	mid S-phase^#^ N (% ± SE)^§^	late S-phase^#^ N (% ± SE)^§^
Control (GM15851 cells)	S1	SS	244	201	193 (61.3 ± 2.75)	8 (4.3 ± 1.50)	0
		DD	44	32	0	24 (13.0 ± 2.48)	8
		SD	278	249	111 (35.2 ± 2.69)	138 (75.0 ± 3.19)	0
		Others	31	25	11 (3.5 ± 1.03)	14 (7.6 ± 1.96)	0
		Total	597	507	315	184	8
	S2	SS	141	115	105 (47.3 ± 3.35)	10 (3.9 ± 1.20)	0
		DD	108	90	2 (0.9 ± 0.63)	67 (25.9 ± 2.72)	21 (80.8 ± 7.73)
		SD	300	279	108 (48.6 ± 3.36)	166 (64.1 ± 2.98)	5 (19.2 ± 7.73)
		Others	26	23	7 (3.2 ± 1.17)	16 (6.2 ± 1.50)	0
		Total	575	507	222	259	26
	S3	SS	48	30	20 (24.7 ± 4.79)	10 (2.7 ± 0.84)	0
		DD	209	179	1 (1.2 ± 1.23)	142 (38.3 ± 2.52)	36 (90.0 ± 4.74)
		SD	281	264	55 (67.9 ± 5.19)	205 (55.3 ± 2.58)	4 (10.0 ± 4.74)
		Others	24	19	5 (6.2 ± 2.67)	14 (3.8 ± 0.99)	0
		Total	562	492	81	371	40
	S4	SS	22	11	4 (23.5 ± 10.29)	7 (2.1 ± 0.78)	0
		DD	346	263	1 (5.9 ± 5.71)	182 (54.7 ± 2.73)	80 (93.0 ± 2.75)
		SD	183	149	11 (64.7 ± 11.59)	133 (39.9 ± 2.68)	5 (5.8 ± 2.52)
		Others	18	13	1 (5.9 ± 5.71)	11 (3.3 ± 0.98)	1 (1.2 ± 1.16)
		Total	569	436	17	333	86
FRDA (GM15850, GM16227 cells)	S1	SS	311	270	251 (62.1 ± 2.41)	19 (13.9 ± 2.95)	0
		DD	21	13	0	10 (7.3 ± 2.22)	3
		SD	212	201	119 (29.5 ± 2.27)	80 (58.4 ± 4.21)	2
		Others	77	62	34 (8.4 ± 1.38)	28 (20.4 ± 3.45)	0
		Total	621	546	404	137	5
	S2	SS	214	193	178 (55.5 ± 2.77)	15 (8.7 ± 2.14)	0
		DD	49	34	2 (0.6 ± 0.44)	26 (15.0 ± 2.72)	6
		SD	238	221	102 (31.8 ± 2.60)	116 (67.1 ± 3.57)	3
		Others	67	56	39 (12.1 ± 1.82)	16 (9.2 ± 2.20)	1
		Total	568	504	321	173	10
	S3	SS	33	28	19 (67.9 ± 8.83)	9 (2.8 ± 0.92)	0
		DD	239	204	1 (3.6 ± 3.51)	100 (31.1 ± 2.58)	103 (69.1 ± 3.79)
		SD	257	231	5 (17.9 ± 7.24)	192 (59.6 ± 2.73)	34 (22.8 ± 3.44)
		Others	49	36	3 (10.7 ± 5.85)	21 (6.5 ± 1.38)	12 (8.1 ± 2.23)
		Total	578	499	28	322	149
	S4	SS	9	4	3	1 (0.7 ± 0.68)	0
		DD	436	309	0	65 (44.2 ± 4.10)	244 (83.3 ± 2.18)
		SD	144	102	1	70 (47.6 ± 4.12)	31 (10.6 ± 1.80)
		Others	42	29	0	11 (7.5 ± 2.17)	18 (6.1 ± 1.40)
		Total	631	444	4	147	293

# Replication patterns are based on features of the FISH signals of BAC RP11-265B8; S-phase cells are classified according to CldU-labeling. All details in Materials and Methods.

^§^ Percentages and SE of percentages were calculated only if > 10 total cells were observed per each S-phase substage.

When the late replicating sequence at *FRA3B* was considered (S3 Table), less than 15% of the S1-S2 cells carried a pair of replicated *FRA3B* alleles (DD patterns), confirming that this locus is replicating later than a wildtype *FXN* allele. Moreover, no differences were detected between control (GM15851) and mutant (GM15850) cells in all sorted S-phase fractions. Therefore, replication patterns of expanded *FXN* alleles were strictly comparable to that of the late replicating *FRA3B* (Table 1 and S3 Table).

To confirm the biological significance of our observations, we evaluated the replication pattern of a genomic region located about 170 kb downstream the *FXN* locus, and identified by BAC RP11-548B3 (S4C Fig). By analyzing S2 and S3 fractions of the cell samples used before, the same replication pattern was found for this region in normal and mutant cells (S4 Table). This led us to conclude that the shift of replication timing occurring in the presence of an expanded repeat did not involve a wide genomic region.

### A single-molecule view of the replication profile of Frataxin

**Fig 2:**
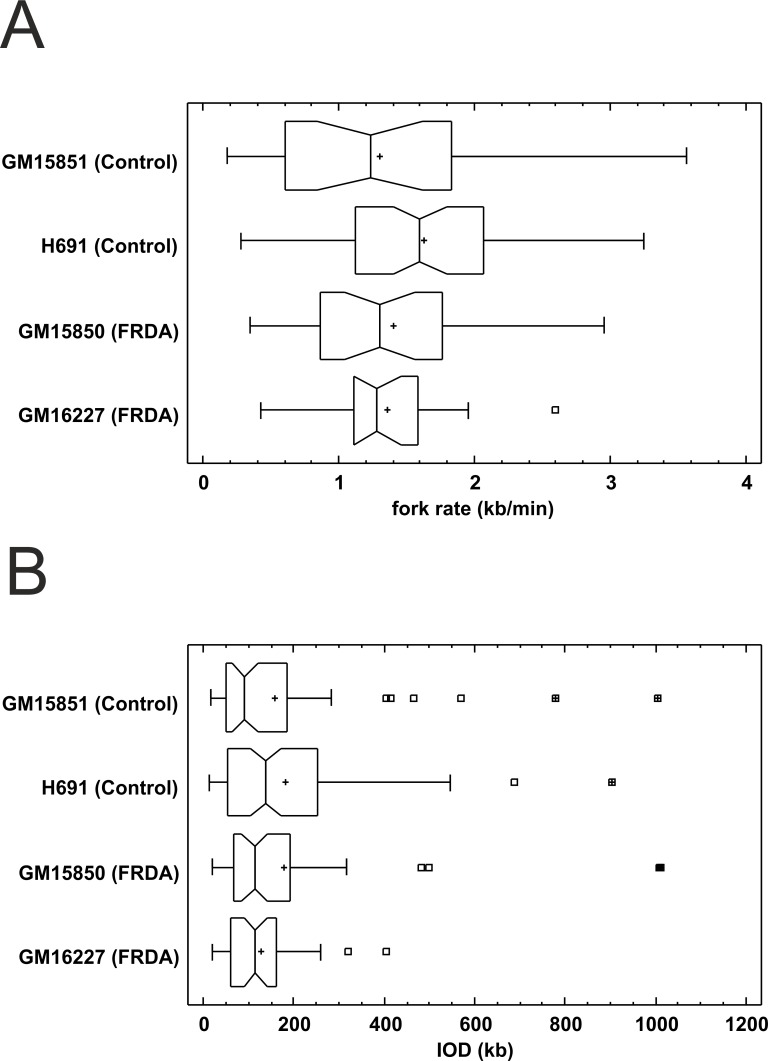
Distributions of fork rates and Inter-Origin Distances (IOD) in a 850 kb region centred around *FXN*. (A) Fork rate distributions observed in two normal (GM15851 and H691) and two FRDA (GM15850 and GM16227) cell lines. (B) IOD distributions observed in the same cell lines as above. No significant differences were detected for both variables described (Kruskal-Wallis non-parametric test).

The replication profiles of normal and mutated *Frataxin* alleles were evaluated in the endogenous genomic context, by monitoring origin firing and replication fork dynamics within a 850 kb region centred on the *FXN* gene (S4 Fig). According to the estimated fractions of replicating molecules, having values higher than 50%, mutant and normal cell lines displayed comparable replication activity within the region (Table 2). At least 100 replication forks were scored and classified per each cell sample (Table 2). Fork rates and Inter-Origin Distances were comparable in normal and mutant cell line, as confirmed by the Kruskal-Wallis non-parametric test, which returned not significant results (Table 2; Fig 2).

**Table 2:** Replication profile of the 850 kb genomic region harboring *Frataxin*.

		GM15851	H691	GM15850	GM16227
		Control	Control	FRDA	FRDA
**Replicating molecules**	Total molecules	61	124	124	63
	Molecules with replication tracks	39	80	70	42
	Fraction of replicating molecules (%)	63.9	64.5	56.5	66.7
**Replication forks**	Total	122	203	151	101
	Unidirectional forks (N)	23	53	49	26
	Unidirectional forks (%)	18.9	26.1	32.5	25.7
	Paused/arrested bidirectional forks (N)^ç^	26	29	21	18
	Paused/arrested bidirectional forks (%)	21.3	14.3	13.9	18.0
	Asynchronous bidirectional forks (N)	8	6	11	1
	Asynchronous bidirectional forks (%)	6.6	3.0	7.3	1.0
**Fork rate (kb/min)**^**§**^	Mean ± SE	1.30 ± 0.166	1.63± 0.090	1.40 ± 0.123	1.36 ± 0.114
	Median	1.23	1.60	1.30	1.29
	N	24	56	29	18
	Min	0.18	0.28	0.35	0.42
	Max	3.56	3.25	2.96	2.60
**Inter-Origin Distance (kb)**	Mean ± SE	156.9 ± 25.63	181.7 ± 18.92	176.9 ± 31.38	126.4 ± 13.22
	Median	90.8	137.6	113.2	115.0
	N	54	78	45	40
	Min	16.6	11.1	19.4	19.0
	Max	1005.7	904. 5	1010.3	403.9

^ç^ Replication forks with bilateral pause/arrest are calculated as a single event, because correlated to the firing of one origin.

^§^ Only bidirectional forks (asynchronous and paused/arrested forks not included).

**Fig 3:**
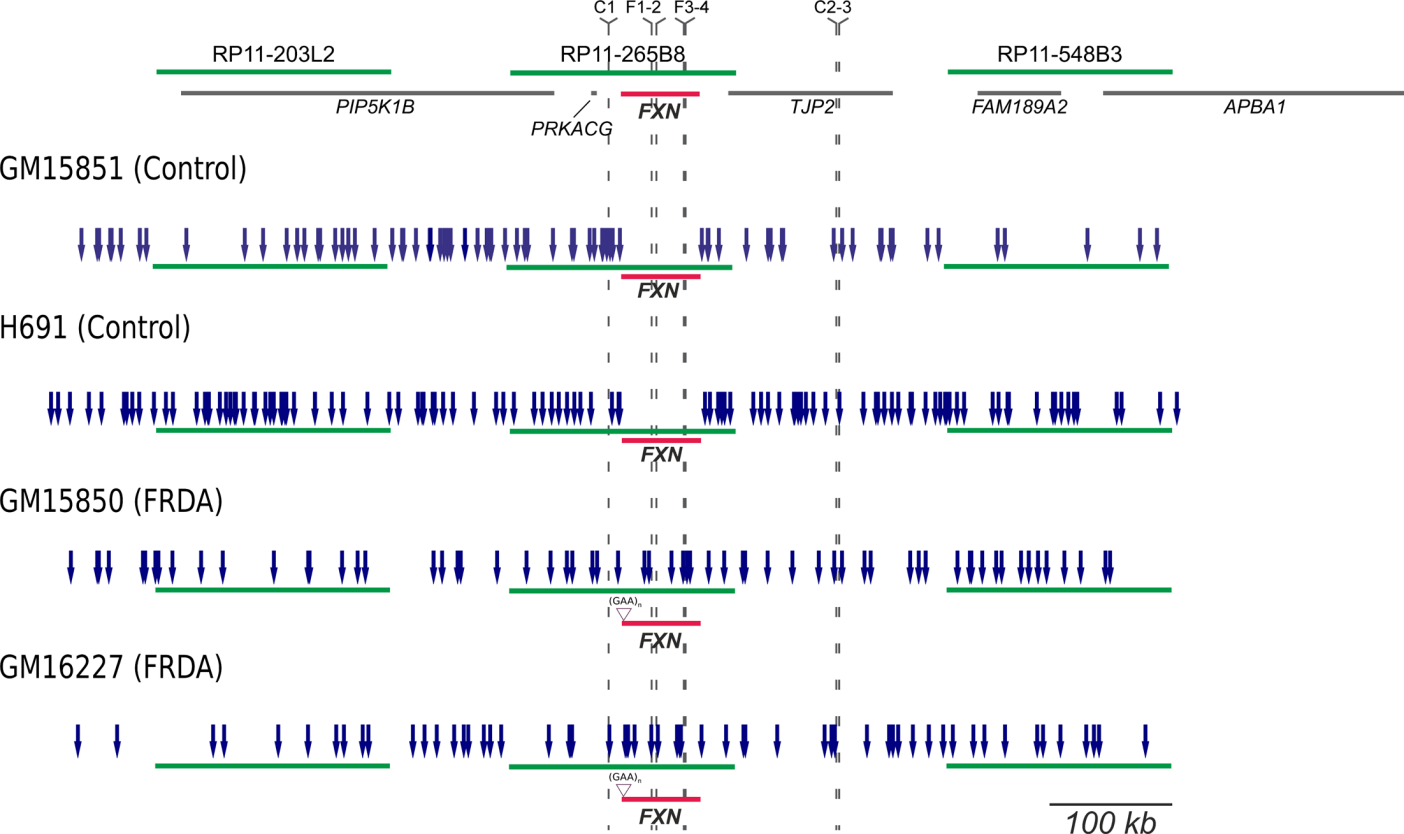
Synoptical view of the initiation events observed in a 850 kb region surrounding *FXN*. Top: the region investigated in the study. Green lines represent the three BAC clones used as probes, gray lines display genes, *FXN* is highlighted in red. The positions of primer sets used in the Short Nascent Strand (SNS) abundance assay are indicated in the map. In the four following schemes, blue arrows indicate the position of each bidirectional origin identified within normal (GM15851, H691) and mutant (GM15850, GM16227) alleles, relatively to the three probes (green) and the *FXN* gene (red). The position of the GAA-repeat expansion in the mutated alleles is also displayed.

**Fig 4:**
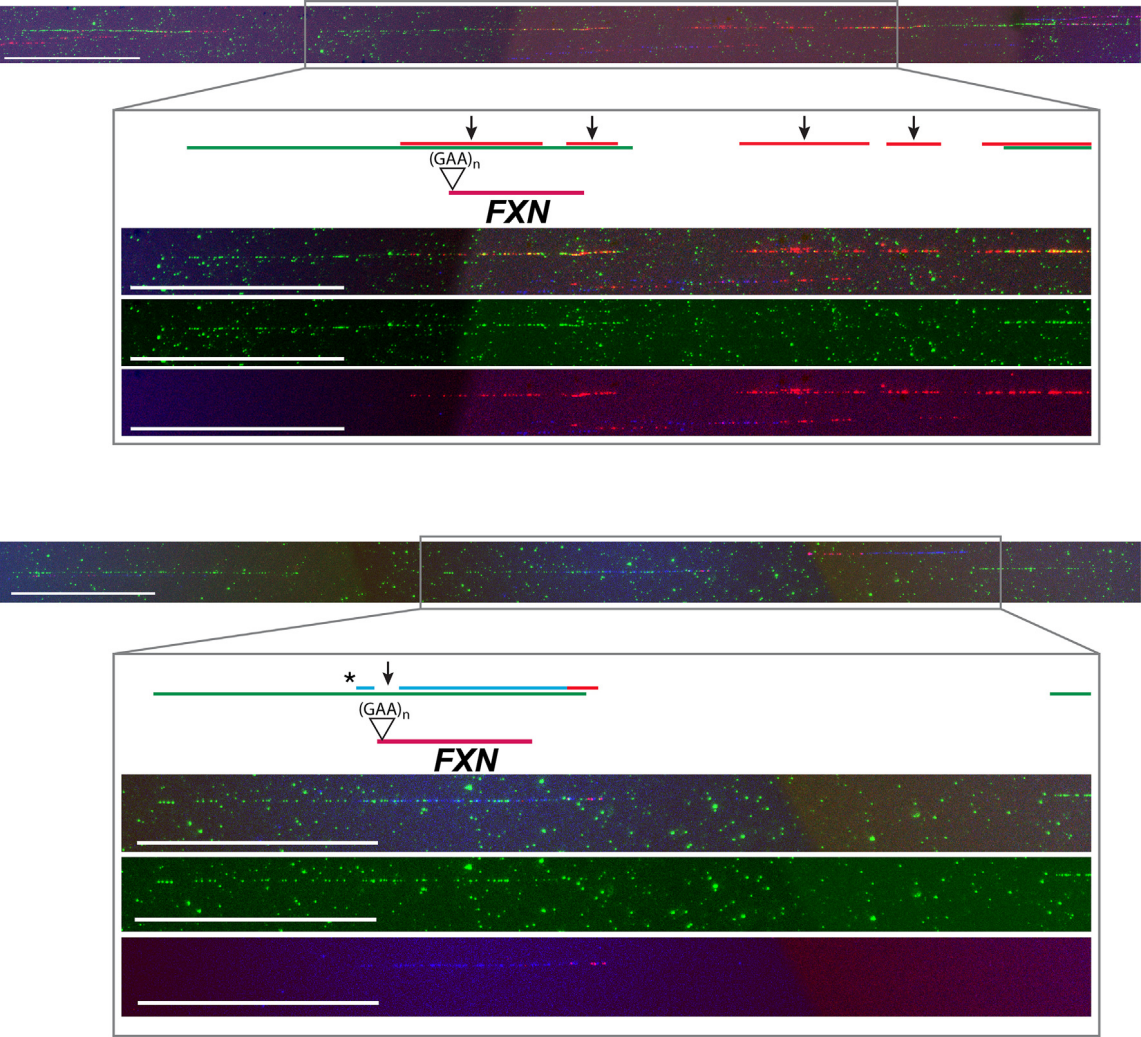
Two representative images of the *FXN* locus as detected by FISH and molecular combing. Three probes (green) are used to detect the 850 kb region harboring *FXN* (red). The position of the GAA-repeat expansion in the mutated alleles is also displayed. Replication tracks are visualized in blue (IdU) and red (CldU), arrows indicate origin positions, the asterisk corresponds to a paused/arrested fork. The two images refer to S8 Fig, molecules 41 and 5 respectively. The region identified by BAC RP11-265B8, where *FXN* maps, is enlarged to allow a better visualization of origin firing and a sharp interpretation of the replication signals. For each enlargement, the first frame corresponds to the merged image, the second frame shows the probes in green fluorescence, the third one displays the blue and red tracks coinciding with the replicative patterns. Calibration bar = 100 kb.

It is accepted that activation of mammalian replication origins does not occur at steady genomic positions [5,6]. In agreement, within the investigated region origin firing occurred with wide molecule-to-molecule variability. The results obtained per single molecule and per each cell line are shown in S5–S8 Figs. A synoptical view showing the position of all the bidirectional origins mapped for the four cell lines investigated is shown in Fig 3. Looking at the whole genomic region delimited by the three BAC probes, changes in origin choice and a differential distribution of activated bidirectional origins were detected in mutated versus normal cells (Fig 3). Dealing with each probe separately, we could appreciate that in the mutated alleles origin choices changed both upstream and downstream *FXN* (Fig 3). However, the most intriguing differences among samples emerged when focusing on the region we are more interested in, the central BAC RP11-265B8 harboring the *FXN* gene. In the normal cell line GM15851, 18 replicating molecules had at least one bidirectional origin firing within that region, 20 origins were mapped in total, but none of them fired inside the *FXN* gene (Fig 3, S5 Fig). The same pattern was found in our second control, the H691 cells: inside the region identified by BAC RP11-265B8 we detected 27 molecules with replication tracks, 16 of them carrying at least one bidirectional origin. No origin fired within the *FXN* gene, although 20 bidirectional origins were detected outside its sequence (Fig 3, S6 Fig). By considering the orientation of the replication forks running through the short GAA-repeat it appeared that this sequence was prevalently, but not exclusively, the template for the lagging strand. Remarkably, in both mutant cell lines several molecules showed one origin firing within the *FXN* allele with the expanded GAA-repeat (Figs 3 and 4; S7–S9 Figs). In particular, in GM15850 cells we found 14 molecules showing at least one bidirectional origin in the region identified by BAC RP11-265B8, for a total of 19 origins mapped within this genomic sequence (Fig 3, S7 Fig). Seven of these origins, each of them firing in an independent molecule, were located within the *FXN* gene in different positions (Fig 3, S7 Fig). A similar profile was seen in GM16227 cells, where 17 bidirectional origins (from 11 molecules) were mapped within the central BAC RP11-265B8, and 9 of them (from 9 diverse molecules) were located in different positions of the *FXN* gene (Fig 3, S8 Fig). In consequence of dormant origin activation within the *FXN* gene sequence, the proportion of forks replicating the GAA-repeat from a downstream origin, and therefore from the leading strand, becomes higher than in the wildtype allele (S5–S8 Figs).

**Fig 5:**
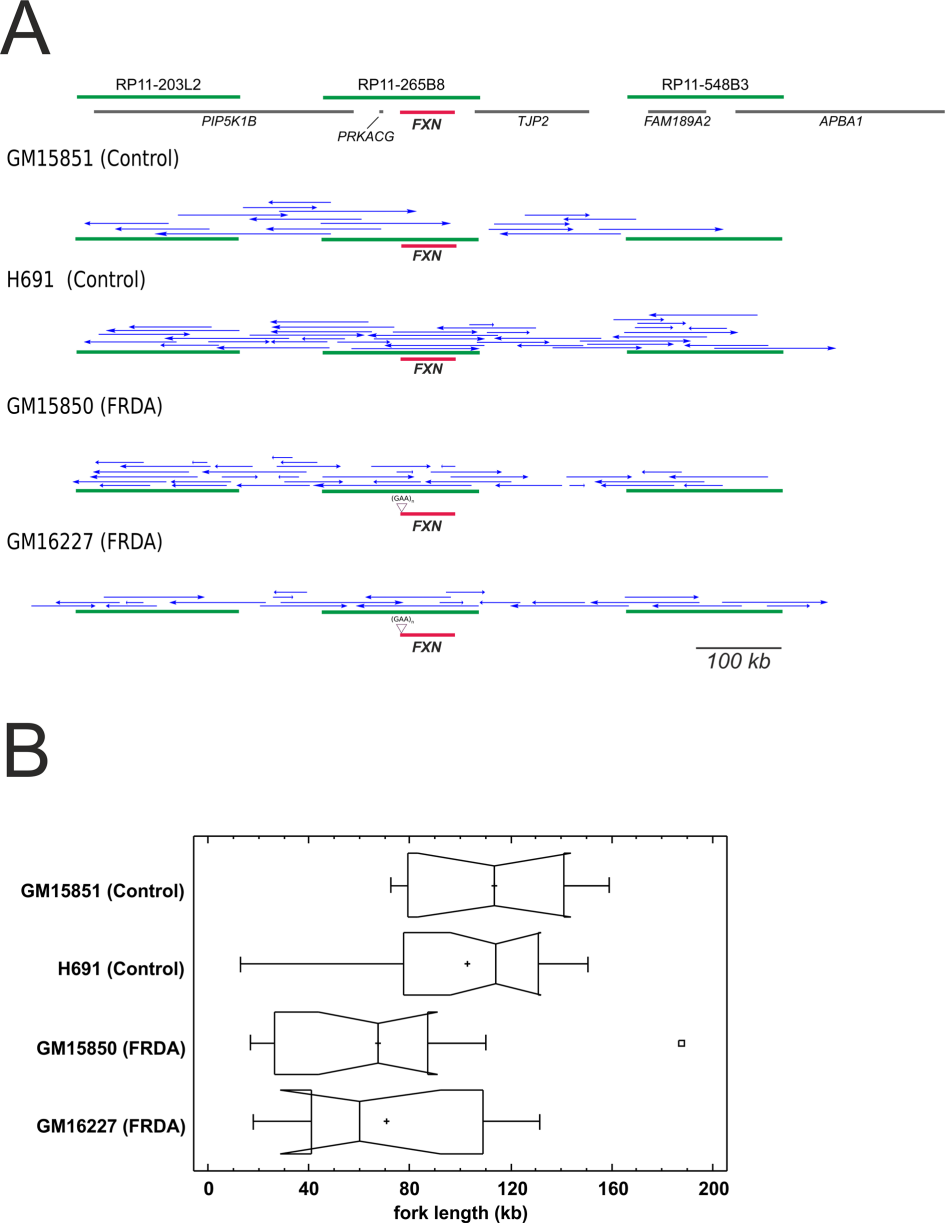
Position, direction and extension of the replication forks with unidirectional pattern observed in the genomic region under study. (A) The region investigated with green lines representing the three BAC clones used as probes, gray lines displaying genes, *FXN* (highlighted in red). In the following schemes, unidirectional fork progression within normal (GM15851, H691) and mutated (GM15850, GM16227) alleles is represented by blue arrows relatively to the three probes (green) and the *FXN* gene (red). The position of the GAA-repeat expansion in the mutated alleles is also displayed. (B) Length distributions of the unidirectional running forks observed in normal and mutant cell lines, in the region including the central BAC and the flanking probe-to-probe distances. There is a significant length reduction in FRDA cells with respect to normal ones (P < 0.01, Kruskal-Wallis non-parametric test).

Replication forks with unidirectional progression were observed in proportions ranging from 19 to 32.5% in the different cell lines (Table 2). In all cell lines, they appeared evenly distributed along the genomic region investigated but a prevalence of short unidirectional forks was noted in FRDA cells compared to the average length detected in normal ones; this difference is particularly evident in the central segment that is harboring the *FXN* gene. Since the actual origin position cannot be defined when forks run unidirectionally, it is not correct to calculate their speed. Hence, we measured the length of unidirectional tracks entirely running in the region including the central BAC and the flanking probe-to-probe distances (Fig 5A). Statistically significant difference was detected among the four distributions, indicating the presence of a marked length reduction in FRDA cells when compared to the normal cell lines GM15851 and H691 (Fig 5B, P< 0.01, Kruskal-Wallis non-parametric test). Average lengths with standard errors were respectively: 113.7 ± 12.41 kb in normal GM15851 cells, 102.8 ± 8.56 kb in normal H691 cells, 67.5 ± 9.81 kb in FRDA GM15850 cells, 70.5 ± 11.83 kb in FRDA GM16227 cells (Fig 5B). In addition, according to the coefficient of variation (CV), unidirectional fork length measures are less dispersed in control cells (CV about 35%) than in FRDA ones (CV about 60%). Length distributions of unidirectional forks were significantly different in FRDA and control cells also when the whole panel of unidirectional forks was evaluated by Kruskal-Wallis test (P < 0.005). In this case the average lengths with standard errors were respectively: 115.9 ± 9.02 kb in normal GM15851 cells, 103.6 ± 5.67 kb in normal H691 cells, 76.9 ± 5.43 kb in FRDA GM15850 cells, 84.3 ± 7.30 kb in FRDA GM16227 cells. The magnitude of the CVs associated with the distribution of unidirectional forks in the whole 850 kb region remain higher in FRDA cells (although CVs decrease to values of about 45%) than in the controls (about 35%).

**Fig 6:**
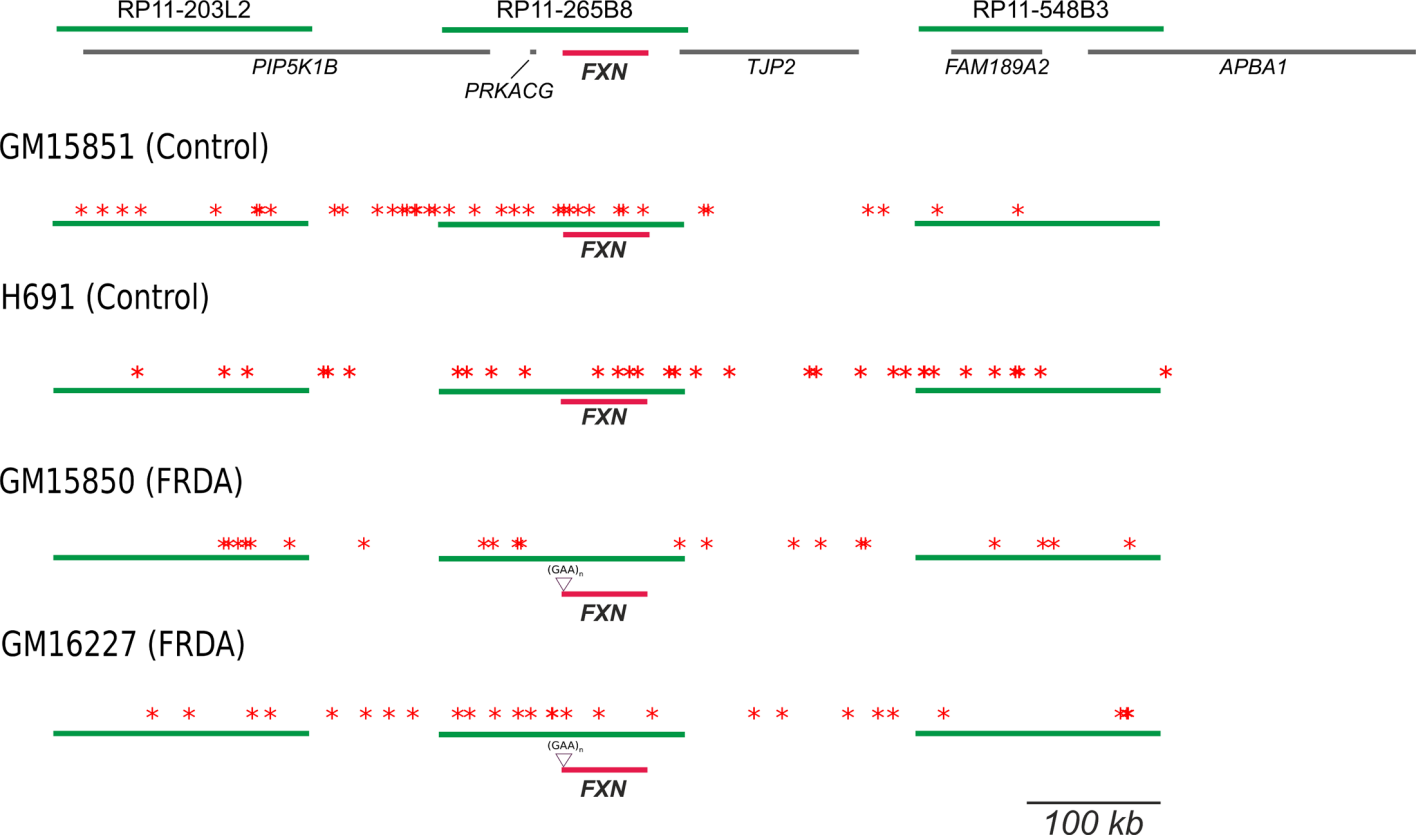
Synoptical view of the events of pause/arrest of the replication fork recorded in the genomic region under study. The region investigated is shown on the top: green lines represent the three BAC clones used as probes, gray lines display genes, *FXN* is highlighted in red. In the four following schemes, each red asterisk indicates a block in fork progression within normal (GM15851, H691) and mutated (GM15850, GM16227) alleles, relatively to the three probes (green) and the *FXN* gene (red). The position of the GAA-repeat expansion in the mutated alleles is also displayed.

Finally, several events of pause/arrest of the fork were observed within the *FXN* locus and in the adjacent sequences. Frequent events of pause/arrest of the fork were detected in proximity of the short repeat in the GM15851 cells, while less intense occurrence of pause/arrest of the fork was found in H691 cells as well as in both mutant cell lines at the position of the long GAA-repeat (Fig 6).

Together, these data indicate that a passive modality of replication is favored within the normal *FXN* sequence, in which the short GAA repeat is the prevailing template for the lagging strand synthesis. In the presence of an expanded repeat, several changes of the replication profile, including recruitment of additional origins within the gene, widespread changes in origin choice, a differential distribution of unidirectional forks, provide the basis for assuring the completeness of DNA replication. In consequence of the activation of dormant origins in the expanded alleles, a switch of the direction by which the replication forks proceed through the GAA-repeat is frequently observed with respect to the normal sequence.

### Short Nascent Strand (SNS) abundance assay

**Fig 7:**
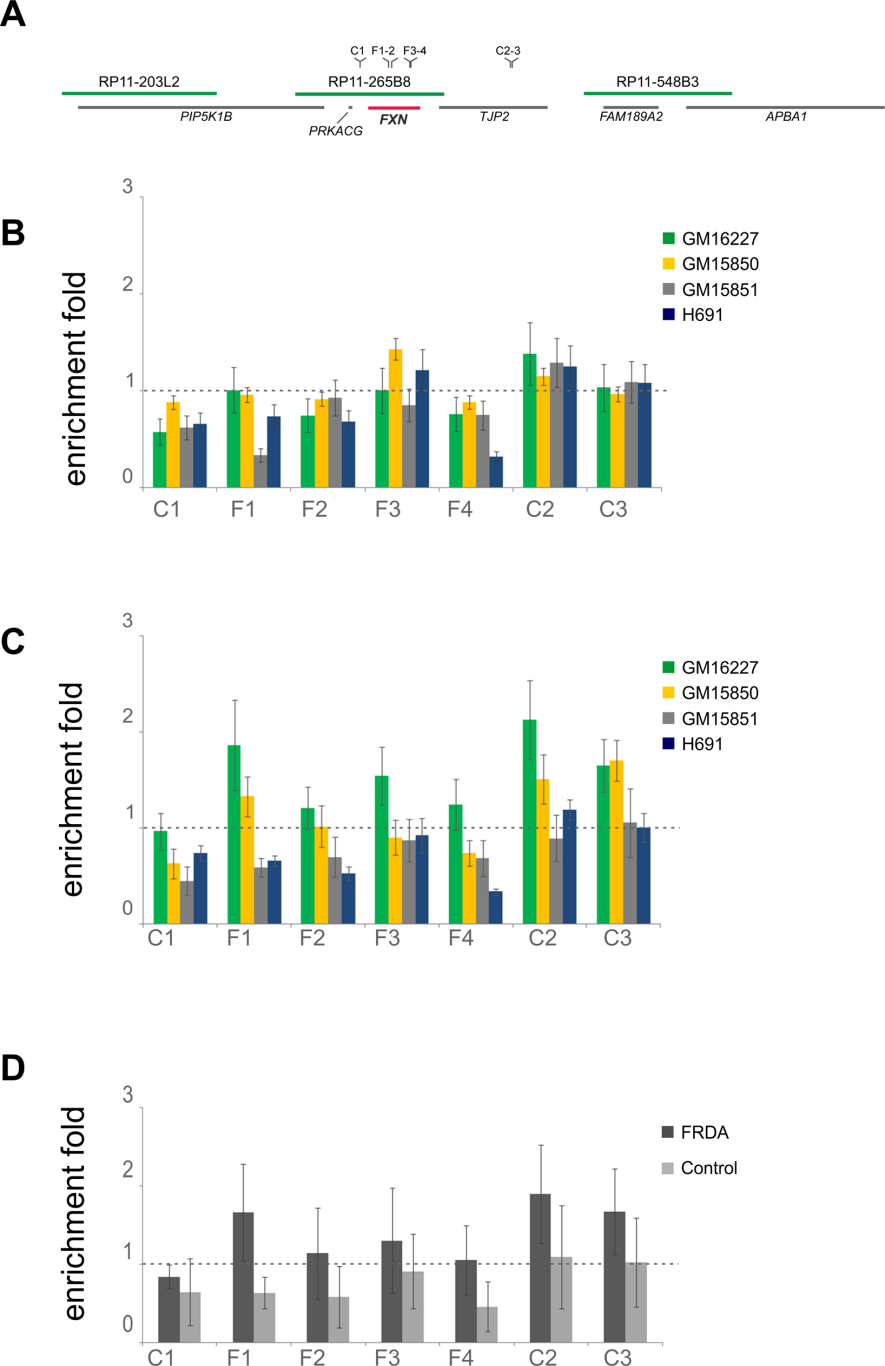
SNS abundance assay results. (A) The map position of primer sets used in the Short Nascent Strand (SNS) abundance assay. Green lines represent the three BAC clones used as probes in molecular combing analyses; gray lines display genes, *FXN* is highlighted in red. (B) Abundance of *FXN* sequences in nascent DNA from two independent isolation experiments for each cell line. The mean quantities of SNS at *FXN* region (primers F1-F4) were normalized to the average quantities determined in the control sites on chr. 9 (primers C1-C3). (C) Abundance of SNS at each primer set on chr. 9 was normalized to *LB2C1*, and further corrected according to the enrichment at the *LAMIN B2* origin, which was set as threshold. (D) Abundance of SNS at each primer set on chr. 9 is shown as pooled data for FRDA (GM16227, GM15850) and control cells (GM15851 and H691); the analysis was performed as in C. Error bars are standard errors of the mean.

The pattern of origin activation in FRDA versus control cells was investigated also by the Short Nascent Strand (SNS) abundance assay and quantitative real-time PCR. To carry out the assay under optimal conditions an origin-free region should be used to normalize the SNS amounts obtained per each primer set [38–40]. Based on our molecular combing data (Fig 3), initiation events are widespread within the 850 kb sequence harboring the *FXN* gene and an origin-free region shared by the four cell lines cannot be firmly identified. Hence, qRT-PCR quantities were normalized versus an origin-positive sequence, a validated alternative approach to analyze SNS abundance experiments [40,41]. Two positions with recurrent pattern of origin activation among the four cell lines, located upstream and downstream the *FXN* gene respectively, may be inferred by the molecular combing analysis and were chosen to design primer sets C1-C3 (S5 Table, Figs 3 and 7A). Concerning the *FXN* gene, by using four primer sets (F1-F4, S5 Table, Figs 3 and 7A) we analyzed two sites downstream the GAA repeat. The enrichment fold at the *LAMIN B2* origin [38,39], calculated as a quality control of each SNS isolation experiment, ranged 5–119 (two independent experiments for each cell line). These values were used to set the threshold to estimate the SNS enrichment in FRDA and control cells as described in Material and Methods.

When the average quantity of SNS in the control regions C1-C3 on chromosome 9 (representing initiation zones) were used as the normalizing factor for the values estimated within the *FXN* gene (F1-F4), no differential patterns were detectable between FRDA and control cells (Fig 7B). Normalization of data versus a sequence with origins can produce a flattening effect and the background noise could be prevalent over true differences in origin activation, especially when dealing with low-efficient events as in this case [42]. To overcome this limitation, which could be responsible for the lack of differentiation visible in Fig 7B, we normalized SNS quantities to the non-origin site *LB2C1*. This value was further corrected by the mean enrichment of *LAMIN B2* origin, estimated for each cell line, as described in Materials and Methods. Although no specific trends emerged when considering each cell line separately (Fig 7C), by pooling data of FRDA or normal cell lines it appeared that SNS quantities detected in normal cells within the *FXN* gene (primers F1-F4) remained under the threshold, while the threshold was reached at the control sites (C1-C3) in all cell lines and at *FXN* in FRDA cells (Fig 7D). This differential response is coherent with a lack of initiation events within the normal *FXN* alleles, but taking together the data shown in Fig 7 it must be concluded that the SNS abundance assay is not sensitive enough to confirm the activation of dormant origins during the replication of *FXN* expanded alleles.

## Discussion

In this study we defined the replication program of the *FXN* gene in human cells, providing for the first time a wide view of origin firing and fork progression within an endogenous genomic context harboring an expanded GAA-repeat. In comparison to the normal *FXN* sequence, we found an altered replication timing of the mutated alleles. According to our results, the replication of expanded *FXN* alleles is slowed or delayed during the first half of the S-phase as compared with the wildtype sequence, while a normalization of this effect can be inferred in the second part of the S-phase.

By evaluating the replication profile of normal cells by molecular combing we found that *FXN* is passively replicated from incoming replication forks. Indeed, origins were never observed within the normal *FXN* sequence, both in this study and in our exploratory analysis of primary human lymphocytes from a healthy subject [14]. Changes in origin choice occurred even several kb upstream and downstream the expanded GAA-repeat, and the most relevant effect was the activation of origins downstream the GAA-expanded repeat, which can be considered dormant origins recruited to assure the replication of the mutated allele. By looking at the number of bidirectional origins per replicating molecule in the region identified by BAC RP11-265B8 (1.11 for GM15851, 1.25 for H691, 1.36 for FRDA GM15850, 1.55 for FRDA GM16227), the trend suggests an enhanced firing associated with the presence of the GAA-repeat expansion, indicating that the activation of the dormant origins does not substitute the initiations occurring in the normal alleles, while they are additional events. The activation of dormant origins at *FXN* has important implications to achieve replication of this gene, because the number of forks firing downstream the GAA-repeat is increased in mutant cells (S7 and S8 Figs) than in normal ones (S5 and S6 Figs). This implies that while in normal cells the GAA-repeat is prevalently located in the lagging strand template, in mutant cells the expansion is often in the leading strand template. This would be in agreement with the predictions of the origin-switch model for trinucleotide repeat instability [17–19], which was recently demonstrated to conform to the case of the CGG-expansion at the *FMR1* locus [20].

The activation of dormant origins is a rare and stochastic event occurring when cells are exposed to replication stress conditions, in order to respond to fork slowing and stalling [43–45]. Differently from the majority of published works in this field [1,46,47], in this study we observed the occurrence of a physiological event restricted to a narrow genomic region. Therefore, the activation of dormant origins at *FXN* is not comparable to an induced massive response as observed when cells are treated with DNA replication inhibitors. Indeed, looking at S7 and S8 Figs. the frequency of dormant origin firing, estimated on the total number of replicating molecules, ranged 14–21%. Moreover, when a dormant origin was found to be activated within *FXN*, it was never associated with additional dormant origins and its location was not restricted to a steady position within the gene. Thus, firing of dormant origins is a peculiar feature of the *FXN* expanded allele, but the event occurs stochastically within the gene and among cells. Detecting such events as enrichment in origin activity through application of the SNS abundance assay is challenging. Moreover, it has been demonstrated that this approach is weakly effective when dealing with mid-late replicating loci [48–51] as in the case of the *FXN* locus, although it is considered accurate/stringent in the characterization of efficient origins, which are activated in early replicating regions. In view of the above considerations it is not surprising that only a weak trend was observed by applying the SNS abundance assay to the *FXN* locus (Fig 7D). However, these results are consistent with the molecular combing data supporting the activation of dormant origins within *FXN* in FRDA cells.

While this manuscript was under revision, data were published by applying a novel approach (OK-Seq), based on Okazaki fragment sequencing, which provides a global description of the replication landscape in a normal lymphoblastoid cell line (GM06990) and in HeLa cells [6]. OK-Seq data clearly indicate that *FXN* is associated with a termination region bordered by two initiation zones located upstream and downstream the gene (displayed as b and c in S10 Fig) in strict agreement with the replication profile obtained by molecular combing for the normal cell lines GM15851 and H691. More precisely, according to the model described in [6] the OK-Seq replication profile of the short termination zone associated with the *FXN* gene fits the scenario of forks emanating from the surrounding initiation zones and converging at different positions within the gene body. Additionally, Petryk et al. [6] identified a large termination region delimited by the initiation zones a and b (S10 Fig) which, according to their criteria, corresponds to a cascade of terminations associated with the firing of background origins. Again, this is coherent with our combing data (S5–S8 Figs). Thus, OK-Seq analysis strengthens the evidence of passive replication of the *FXN* gene in the absence of the expanded repeat, demonstrating that our observation can be extended to other cell lines and to diverse cell types. Despite the high resolution and precision of OK-Seq in identifying also broad and disperse initiation/termination zones [6], in the case of the expanded *FXN* allele it would be very hard to demonstrate the isolated, rare and widespread activation of dormant origins, because their firing may be not strong enough to generate a detectable initiation zone (Hyrien O. personal communication). On the whole, we can conclude that single molecule approaches, such as molecular combing, are the most appropriate tool to identify rare and stochastic firing events occurring as a change of the locus-specific replication program. In agreement with this opinion, for testing the reliability of genome wide approaches (e.g. ChIP-Seq) Dellino and Pellicci [42] recommend the application of single molecule techniques, because they provide relatively high-resolution origin maps in a large number of DNA molecules within the chromosomal region of interest [52–54].

In this study, a marked reduction of the average length of unidirectional running forks was observed in FRDA cells by molecular combing (Fig 5). Length values were also more broadly distributed in FRDA cells than in controls, in particular in the region delimited by the central BAC. We previously demonstrated that unidirectional forks are frequently detected in human cells by molecular combing and their frequencies ranged in the same interval observed here [14]. The biological meaning of the unidirectional forks is still unraveled, however in this case the length reduction observed in FRDA cells could be regarded as an additional effect of the replication impairment associated with the expanded GAA-repeat.

Using human cell lines from FRDA patients we were able to follow up the replication behavior of sequences carrying 630 to 1030 GAA-repeats, which are rather long arrays with respect to those evaluated in transfected/engineered cells. In these cell lines, we found that the functional loss of Frataxin does not have major effects on cell proliferation activity, and it is not associated with global changes during the replication process (S1 Fig). In agreement with the published results [10–12,32], here we found that long stretches of GAA-repeats do not represent a strong impediment for the replication process. Indeed, although a shift of the replication timing was detected in mutated *FXN* alleles with respect to the normal ones, a rapid normalization of this effect occurs in the second half of the S-phase (Table 1). Furthermore, our molecular combing results suggest that in patients’ cells the replication of the *FXN* gene is completed through the activation of origins that would not fire under normal conditions. This can be viewed as a rescue mechanism, as it is well known that in mammalian cells fork stalling related to a replication impediment may be solved by activating adjacent dormant origins [4,5].

Previously, different strategies were applied to evaluate the replication of GAA-repeats in vivo. By cloning stretches of different length in yeast plasmids [10], replication stalling was detected in long tracts ranging the size of premutated or mutated human alleles, while it was not observed in the presence of short (< 40) repeats. In consequence of fork stalling, inhibition of the fork progression, in the order of about 1.5 times, was reported [10]. More recently, a model was developed with a SV40-based plasmid transfected in human cells [11]. DNA replication could progress through expansions in the range of 33–90 GAA repeats, although several abnormal intermediates were found [11]. Transient pausing of the replication fork was detected at GAA-repeats longer than 66 trinucleotides, and in the case of longest tracts (> 90) fork reversal was found to be associated with fork pausing [11]. Moreover, both in yeast and transfected human cells it has been demonstrated that the most significant increase of fork pausing occurs when the GAA-repeat is located in the lagging strand template [11,55]. In the present study, we recorded several and widespread pause/arrest events in the 850 kb region harboring *FXN*. According to our previous results [14], in human cells these patterns often represent normal events of the replication program and DNA combing does not allow to distinguish a physiologically pausing fork from an event caused by a replication impairment. On the other hand, because their position can be mapped precisely and trinucleotide repeats could represent an obstacle for the progression of the replication forks, the preferential localization of pause/arrest events at the GAA-repeat can be checked. Recurrent events of pause/arrest of the fork were recorded in proximity of the short repeat in the GM15851 cells but not in the second control line and FRDA cells. To explain these differences the global response to the replication impairment associated with the presence of the GAA-repeat must be considered in its complexity. Here we demonstrated that in FRDA cells a major role is played by activation of dormant origins, and changes in unidirectional fork progression may be also involved. It is noteworthy that both activated dormant origins and unidirectional running forks are long-lasting replication patterns, while in most of the cases fork pausing are transient events; this feature has been reported also for the GAA-repeat by Follonier et al. [11]. In this frame, the activation of the dormant origins could be the most evident and easily detectable effect that can be ascribed to the presence of the expanded trinucleotide repeat. The reduction of the length of the unidirectionally running forks in FRDA cells is a second evident effect associated with the expanded GAA-repeat. In contrast, the chance to detect paused replication forks is affected by their transient nature. Moreover in FRDA cells the activation of dormant origins acts a safeguarding mechanism assuring the replication of the *FXN* gene. The consequence is that the number of forks running in the opposite direction through the GAA-repeat is increased (S7 and S8 Figs) and this sequence is replicated preferentially from the leading strand template, an orientation not frequently involved in fork pausing according to model systems [11,55]. Thus, in spite of the weak evidence available from this study, the detection of fork arrest events in proximity of the short GAA-repeat in GM15851 control cell line must be taken into further consideration as a possible impact of the short non-pathological GAA-repeat on fork progression. Interestingly this result is in line with that obtained by a single molecule replication assay monitoring about 350 kb at the *FMR1* locus, where fork stalling at the CGG-repeat was found also in normal cells [20].

By molecular combing, we had the opportunity to monitor the *FXN* gene together with the surrounding genomic segment and a fine picture of the events associated with the replication of DNA tracts carrying trinucleotide repeats has been provided. In the present study, *FXN* replication profiles were evaluated on differentiated cells; understanding if the occurrence of origin-switch near the GAA-repeat may cause trinucleotide expansion in FRDA families, or it is instead a consequence of the expansion and/or the associated epigenetic phenomena, remain to be unraveled by further investigations.

## Materials and Methods

### Cell cultures and growth curves

Epstein Barr virus-transformed lymphoblastoid cell lines from two unrelated FRDA patients GM15850, (carrier of alleles with 650 and 1030 GAA-repeats respectively) and GM16227 (carrier of alleles with 630 and 830 GAA-repeats respectively), and from the healthy brother GM15851 of patient GM15850, were obtained by the Human Genetic Cell Repository of the Coriell Institute (USA). The H691 cell line is an Epstein Barr virus-transformed lymphoblastoid cell line established from a young healthy male adult. Cells were grown in RPMI 1640 medium supplemented with 15% foetal bovine serum (EuroClone, Italy) and penicillin/streptomycin antibiotics (Gibco, Life Technologies). The estimated duplication times range between 29–32 h for all cell lines.

### Genotyping and RT-PCR

50 ng of genomic DNA and 10 pg of BAC clone RP11-265B8 were amplified in long-range PCR reactions (QIAGEN Long range PCR kit, Qiagen) using a primer set specific for the amplification of the GAA-repeat (forward: 5’-GGAGGGATCCGTCTGGGCAAAGG-3’, reverse: 5’-CAATCCAGGACAGTCAGGGCTT-3’; normal amplicon length: 1,5 kb) [21]. For the expression analysis total RNA was isolated using the RNeasy mini kit (Qiagen). 1 μg of RNA was retrotranscribed (EuroScript M-MLW Reverse Transcriptase (RNase H-), Euroclone) and 2 μl of cDNA were used as template for PCR. The primer pairs used for *FXN* transcript were forward: 5’-CCTTGCAGACAAGCCATACA-3’, reverse: 5’-GGTCCACTGGATGGAGAAGA-3’ (amplicon length: 153 bp). *GAPDH* was chosen as a normalizing gene and its transcript amplified by forward: 5’-CCTCAACGACCACTTTGTCA-3’, reverse: 5’-TTCCTCTTGTGCTCTTGCTG-3’ (amplicon length: 143 bp).

### Proliferation assays and FACS sorting

Cell cycle distribution of GM15851, GM15850 and GM16227 cell lines was monitored by flow cytometry (FACS Canto II; Becton Dickinson) after propidium iodide staining according to standard protocols, and analyzed with the Cell Quest software (Becton Dickinson).

Cell sorting was carried out in agreement with [37] using a BD FACSAria (BD Biosciences). Briefly, per each experiment 150 x 10^6^ cells, pre-labeled with a 30 min pulse of 100 μM 5-Chloro-2’-deoxyuridine (CldU; Sigma-Aldrich), were harvested and prepared for FACS analysis. On the basis of the observed cell cycle distribution, intervals were set in order to collect four fractions of identical size spanning the entire S-phase. The purity of each S-phase fraction was assessed at the end of the experiment.

### Interphase FISH analysis

Replication timing of normal and expanded *FXN* alleles was determined by interphase FISH in asynchronous and sorted cell line populations. Slides were prepared by using Cytospin 3 (Shandon Scientific Limited, UK) following a standard procedure. BAC clones were obtained by Children's Hospital Oakland Institute (CHORI, USA); BAC DNA was labeled with biotin-16-dUTP by nick translation kit (Roche Biochemicals).

Slides were post-fixed in 3:1 ethanol/acetic acid solution on ice, digested with 5 μg/ml Pepsin in 0.01 M HCl, pH 3.0 at 37°C for 10 min, and dehydrated in 70%, 90% and 100% ethanol. After denaturation at 72°C for 4 min in 70% formamide, 2X SSC pH 7.0 slides were dehydrated in ethanol solutions. The probe mix (100 ng of BAC DNA, 50X human Cot1 DNA (Invitrogen, LifeTechnologies) 5X Salmon Sperm DNA (Invitrogen, LifeTechnologies)) was denatured 10 min at 70°C and pre-annealed at 37°C for 90 min. Per slide, 10 μl were applied under a 22x22 mm coverslip; hybridisation was carried out overnight in a humidified chamber. Post-hybridisation washes were 50% formamide, 2X SSC pH 7.0 at 42°C (3 times), 2X SSC pH 7.0 (3 times). Slides were incubated 30 min at 37°C in 1X blocking solution (Roche Biochemicals) before immunodetection. CldU was detected by a rat monoclonal anti-BrdU antibody specifically cross-reacting with CldU (1:40, Abcam) and a 594 Alexa Fluor-conjugated anti-rat IgG (1:100; Molecular Probes, Life Technologies). At the same time the biotin-labeled probe was detected by 488 Alexa Fluor-conjugated streptavidin (1:300, Molecular Probes, Life Technologies), followed by polyclonal biotin-conjugated anti-streptavidin (1:300; Rockland) and 488 Alexa Fluor-conjugated streptavidin (1:300; Molecular Probes, Invitrogen). Slides were counterstained with DAPI solution (2 μg/ml) in Vectashield Mounting medium (Vector, USA).

### Molecular combing

Exponentially growing lymphoblastoid cells were labeled with two sequential 30 min pulses of 5-Iodo-2’-deoxyuridine (IdU; Sigma-Aldrich) and 5-Chloro-2’-deoxyuridine (CldU; Sigma-Aldrich) (S4 Fig). 1-2x10^5^ cells were immobilized in agarose plugs and incubated overnight at 50°C in 2 mg/ml Proteinase K solution (1% N-laurosylsarcosine, 0.1 M EDTA pH 8.0, 0.01 M Tris-HCl pH 8.0, 0.02 M NaCl). After digestion with β-agarase I (BioLabs), high molecular weight DNA from 1–2 plugs was delivered in 0.1 M MES pH 6.1. According to a standard procedure, DNA combing was performed on silanised surfaces. For further details see [56].

### Single-locus replication analysis

A region spanning 850 kb, identified by the three differentially spaced BAC genomic clones RP11-203L2, RP11-265B8 and RP11-548B3 (Children's Hospital Oakland Institute; CHORI, USA), was used for the single-locus replication analysis (S4 Fig). Probes were biotin-labeled by random priming (BioPrime DNA labeling System, Invitrogen, Life Technologies); the central BAC RP11-265B8, which harbour *FXN*, was also labeled with a custom-made nucleotide mix containing Cy5-AP3-dUTP (GE Healthcare) to allow its identification and orientation also in molecules showing a probe pair instead than the whole probe set. Per slide, 250 ng of each probe were mixed in 20 μl of hybridization solution (50% formamide, 1% N-Laurosylsarcosine, 10 mM NaCl, 2X SSC in BlockAid (Invitrogen, Life Technologies) containing 13X human Cot-1 DNA (Invitrogen) and 10 μg Salmon Sperm DNA (Invitrogen). Denaturation was carried out at 80°C for 10 min. Slides were denatured for 15 min in 1 M NaCl, 0.05 M NaOH, immediately dehydrated in ethanol solutions (70%, 90%, 100%) on ice, hybridized with the probe mix (20 μl under 22x22 mm coverslips) for 19 hours at 37°C in a humidified chamber. Stringency washes were: 3x5 min in 50% formamide, 2X SSC pH 7.0 followed by 3x5 min in 2X SSC pH 7.0. A three-colour scheme of immunodetection was used to localize hybridisation signals together with replication tracks: biotinylated probes were detected in green, whereas IdU and CldU in blue and red, respectively (S4 Fig). Three layers of antibodies were applied (30 min each): in the first one, 488 Alexa Fluor-conjugated streptavidin (1:50; Molecular Probes, Invitrogen) allowed probe detection and two primary anti-BrdU antibodies were used cross-reacting respectively with IdU (2:7; Becton-Dickinson, developed in mouse) and CldU (1:40; Abcam, developed in rat). In the second layer a polyclonal biotin-conjugated anti-streptavidin antibody (1:50; Rockland, USA), a 350 Alexa Fluor-conjugated anti-mouse IgG (1:50; Molecular Probes, produced in goat) and a 594 Alexa Fluor-conjugated anti-rat IgG (1:50; Molecular Probes, produced in donkey) were mixed. In the third layer, 488 Alexa Fluor-conjugated streptavidin mixed with 350 Alexa Fluor-conjugated anti-goat IgG (1:50; Molecular Probes, made in donkey) were used to complete the amplification steps. Cy5-labeled probes do not require amplification of the hybridization signal.

Genome-wide replication of lymphoblastoid cell lines was assessed by IdU and CldU immunodetection, according to the protocol described in [14].

### Image analysis

A motorized fluorescence microscope (Zeiss Axio Imager.M1) equipped with a CCD camera (Photometrix, Coolsnap HQ2) was used for microscope analyses.

Interphase FISH analysis was carried out under a 100X oil immersion objective (N.A. = 1.30) and more than 250 nuclei were scored per each experiment. Replication timing was evaluated according to the observed hybridization signals. Single spots (S) can be referred to unreplicated alleles, whereas duplicated signals (D) to replicated alleles. Thus, nuclei were classified as SS when both alleles were not replicated, SD when only one allele completed replication and DD if both alleles were already replicated. In parallel and by blind analysis, CldU-positive nuclei were recorded and classified according to their fluorescent patterns in early, mid and late S-phase [57]. The hybridization efficiency was calculated by the formula: [SS+SD+DD+1/2(S+D)/total number of scored nuclei] x100.

Molecular combing analyses were performed using a 40X oil immersion objective (N.A. = 1.30). DNA molecules may span several kilobases, therefore adjacent fields were acquired under adequate filter sets, then merged and aligned using Adobe Photoshop CS2 software. Fluorescent signals corresponding to replication tracks and hybridized probes were measured by the Metavue Research Imaging System (Molecular Devices), according to the molecular combing calibration factor (1 μm = 2 kb) and to the magnification features of objectives and CCD camera (1 pixel = 0.16125 μm = 0.3225 kb).

Probe length and probe-to-probe distances were determined in order to orientate molecules and to detect the replication activity within *FXN* genomic region. Moreover, only molecules showing the hybridization of at least two probes were considered informative and were used to calculate the fraction of replicating molecules, as the ratio between the number of molecules displaying replication signals and the total number of observed molecules.

Fork rates, inter-origin distances and replication origin positioning define replication profiles. In order to correctly interpret fluorescent signals, stringent criteria were applied according to those described in details elsewhere [14,58]. Briefly, as genomic DNA was not counterstained, only fluorescent replication signals in a linear array and framed by probe signals were considered, as they belong with maximum confidence to the same single molecule. Replication rates were calculated with complete bidirectional forks only and all other patterns, including forks with unidirectional progression and possible deregulation events, such as asynchronous and paused/arrested forks, were recorded only when upstream or downstream replication tracks supported the presence of uninterrupted filaments. Blue-only tracks emanating from origins fired during the first pulse were interpreted according to the whole replication pattern along the molecule as termination events occurring during the first pulse OR paused/arrested forks. Isolated tracks, not allowing a non-ambiguous interpretation, were excluded from the analysis. More information in [14].

### Short Nascent Strand (SNS) abundance assay and real-time PCR

Short Nascent Strand (SNS) abundance assay [40] was employed with some modifications. 70 × 10^6^ cells derived from the four cell lines (GM15850, GM15851, GM16227, H691) were washed with 1X PBS and collected in 240 μl of 10% glycerol/PBS. 60 μl of each cell sample were lysed for 15 min a denaturating 1.25% agarose gel (50 mM NaOH, 1 mM EDTA), 4°C. The electrophoresis was carried out under the same denaturing conditions for 5–6 h at 30 V, neutralized in 1X TAE and stained with ethidium bromide. DNA fragments 0.5–1.5 kb in length were purified from the gel using a QIAEX II Gel extraction kit (Qiagen). Genomic DNA was purified by phenol-chloroform-isoamylalchol method from cells derived by the same cultures used for the isolation of SNS samples and digested with 0.4 mg/ml Proteinase K and 20 μg/ml RNase A. SNS and genomic DNA were quantified by NanoDrop 1000 (ThermoScientific).

Quantitative real-time PCR was carried out with 0.2 μM of each primer and the Power SYBR Green PCR Master Mix (Life Technologies) in an Applied Biosystems 7500 Real-Time PCR System (Life Technologies). The primer binding sites span the whole *FXN* gene and two regions located upstream and downstream the gene, chosen as controls on the chromosome 9. In addition, origin/non origin sites previously characterized around the *LAMIN B2* gene [38,39] were used as a further control site to test the reliability of the qPCR assay. All primers pairs used are listed in S5 Table and their positions on the *FXN* gene are shown in Figs 3 and 7A. For each primer set, a 6 point standard curve derived from 1:3 serial dilutions of the total DNA of each cell line was amplified in each plate, starting from 4.5 x10^4^ copies of genomic equivalents. Standard curves were run in triplicate, SNS samples were run in five-eight replicas and the mean quantity of genomic equivalents in the short nascent DNA samples was determined in comparison to the correspondent standard curve. Amount of SNS at each primer set was normalized:

1. to the average quantity of SNS estimated in the control regions on the chromosome 9 (primer sets C1-C3) for each cell line.
2. To the non-origin site *LB2C1*, and further corrected according to the enrichment estimated at the *LAMIN B2* origin, used as a threshold to establish the enrichment fold for each primer set in the *FXN* and control regions (primer sets F1-F4, C1-C3).

## Supporting Information

S1 Fig Genotype, transcriptional, and proliferation activity of the cell lines used in this study. (A) The cell lines used in the study; information is provided by the Coriell repository apart from H691 cells; (B) the length of *FXN* alleles in the four cell lines, as evaluated by long-range PCR analysis; (C) transcriptional activity of *FXN* in the four cell lines, as evaluated by semiquantitative RT-PCR; (D) flow cytometry-based cell cycle profiles of the cell lines obtained from the Coriell repository; (E) genome-wide molecular combing analysis in the three cell lines obtained from the Coriell repository.
(JPG)

S2 Fig Pre- and post-FACS sorting cell cycle distribution. FACS sorting experiments aimed to separate cells in consecutive temporal windows of the S-phase (S1-S4). Experiments 3 and 4 represent two independent biological replicates in which GM15851 cells were separated by FACS sorting.
(JPG)

S3 Fig Quality control and reproducibility of the FACS sorting experiments. Before fractionation in four consecutive temporal windows of the S-phase (S5–S8, S2 Figs), cells were exposed to CldU for 30 min immediately before harvesting to confirm the accuracy of cell sorting procedure by CldU-immunodetection. Cells were classified in early, mid, late S-phase according to the observed fluorescent pattern (see F). (A-D) Quality control: data in the graphs were collected during the FISH analyses of *FXN* replication timing (respectively: experiments 1–4 summarized in S2 Fig; raw data in Supplementary Table 2). In all of the experiments each cell fraction is enriched for the expected S-phase stage (error bars = errors of percentages). (E) Reproducibility: average proportions of cells belonging to different stages of the S-phase, as observed in the course of three FISH experiments carried out independently on the same S2 and S3 cell samples (respectively: exp. 2 for GM15850 cells, exp. 3 for GM15851). (F) Examples (left to right) of early, mid, late S-phase nuclei according to the observed fluorescent CldU pattern.
(JPG)

S4 Fig Experimental design and criteria of analysis in molecular combing experiments. (A) The two-pulse labeling scheme for detection of replication forks. In the first pulse IdU is incorporated in the nascent strands and labeled DNA is detected by blue fluorescence; during the second pulse CldU is available for the synthesis of DNA, and labeling is detected by red fluorescence. (B) Examples of normal and altered replication patterns expected in single-locus replication analyses; replication tracks and probes are represented slightly displaced for simplicity. Three probes differentially spaced (D1 and D2) are detected by green fluorescence: the FISH pattern allows us to define the centromere-telomere orientation and the integrity of the molecule (a Cy5-labeled central probe is cohybridized with the biotin-labeled probes, to allow the centromere-telomere orientation when only two hybridization signals can be visualized). Bidirectional origins (o) may be mapped in the middle of the two arms or in the middle of a blue track of a replication fork. Paused/arrested forks (*) may be unilateral or bilateral events, as illustrated. Asynchronous forks (as.) fire from the origin with different rates. Unidirectional forks (unidir.) are identified when a single arm with blue/red pattern is progressing with same orientation than the upstream or downstream track (which is the case represented in this example). (C) The genomic region investigated in this study. Green lines represent three probes covering about 850 kb in the bp interval 68643,187–69477,097 at 9q21. *Frataxin* (*FXN)* is shown in red and the other genes mapping in the flanking regions are shown in grey.
(JPG)

S5 Fig Fork progression and origin distribution in single molecules spanning the region investigated in GM15851 (control) cells. Dotted lines indicate the boundaries of the *FXN* sequence. A scheme of the genomic region is shown in S4C Fig Original reconstructed images are deposited in the Dryad Digital Repository at http://dx.doi.org/10.5061/dryad.f12cg.
(JPG)

S6 Fig Fork progression and origin distribution in single molecules spanning the region investigated in H691 (control) cells. Dotted lines indicate the boundaries of the *FXN* sequence. A scheme of the genomic region is shown in S4C Fig Original reconstructed images are deposited in the Dryad Digital Repository at http://dx.doi.org/10.5061/dryad.f12cg.
(JPG)

S7 Fig Fork progression and origin distribution in single molecules spanning the region investigated in GM15850 (FRDA) cells. Dotted lines indicate the boundaries of the *FXN* sequence. A scheme of the genomic region is shown in S4C Fig Original reconstructed images are deposited in the Dryad Digital Repository at http://dx.doi.org/10.5061/dryad.f12cg.
(JPG)

S8 Fig Fork progression and origin distribution in single molecules spanning the region investigated in GM16227 (FRDA) cells. Dotted lines indicate the boundaries of the *FXN* sequence. A scheme of the genomic region is shown in S4C Fig Original reconstructed images are deposited in the Dryad Digital Repository at http://dx.doi.org/10.5061/dryad.f12cg.
(JPG)

S9 Fig Representative images of replication at *FXN* (RP11-265B8 probe) as detected by FISH and molecular combing in FRDA and control cells. Images are selected enlargements from complete reconstructed molecules (from top to bottom: molecules 33, 42, 15 in S5 Fig, molecule 13 in S6 Fig, molecules 36 and 7 in S8 Fig, molecule 42 in S7 Fig) The probe (green), containing the *FXN* gene (red), and the flanking regions (probe-to-probe distances, S4C Fig) are shown. Replication tracks are visualized in blue (IdU) and red (CldU), arrows indicate origin positions, the asterisk corresponds to a paused/arrested fork, unidirectional forks are indicated. GAA-repeat expansion is also displayed. Calibration bar = 100kb.
(JPG)

S10 Fig Replication fork directionality profiles (RFD) of GM06990 and HeLa cells in the region harboring the *FXN* gene (GRCh37, hg19). (A) RFD profile of the GM06990 lymphoblastoid cell line. Green lines represent the three BAC clones used as probes in molecular combing experiments, gray lines display genes, *FXN* is highlighted in red. Pink bars (a, b, c) represent initiation zones derived by OK-Seq analysis [6]. Two replicas are shown. (B) RFD profile of HeLa cell line. Green lines represent the three BAC clones used as probes in molecular combing experiments, gray lines display genes, *FXN* is highlighted in red. Pink bars (a, b, c, d) represent initiation zones derived by OK-Seq analysis [6]. Two replicas are shown. RFD were downloaded from http://157.136.54.88/cgi-bin/gbrowse/gbrowse/okazaki_ref/.
(JPG)

S1 Table Raw data of the replication timing analysis of *FXN* carried out by interphase FISH. (DOCX)

S2 Table Raw data of the replication timing analysis of *FXN* carried out by interphase FISH after FACS cell sorting. (DOCX)

S3 Table Replication timing of the late replicating sequence *FRA3B* according to interphase FISH after FACS cell sorting. (DOCX)

S4 Table Replication timing of a genomic domain located about 170 kb downstream the *FXN* allele, according to interphase FISH after FACS cell sorting. (DOCX)

S5 Table Oligonucleotide primer sequences. (DOCX)
