# Supplementary figures and images for: The Replication of Frataxin Gene Is Assured by Activation of Dormant Origins in the Presence of a GAA-Repeat Expansion
Source: PLoS Genet. 2016 Jul 22;12(7):e1006201. doi: 10.1371/journal.pgen.1006201 (PMC4957762; doi:10.1371/journal.pgen.1006201)

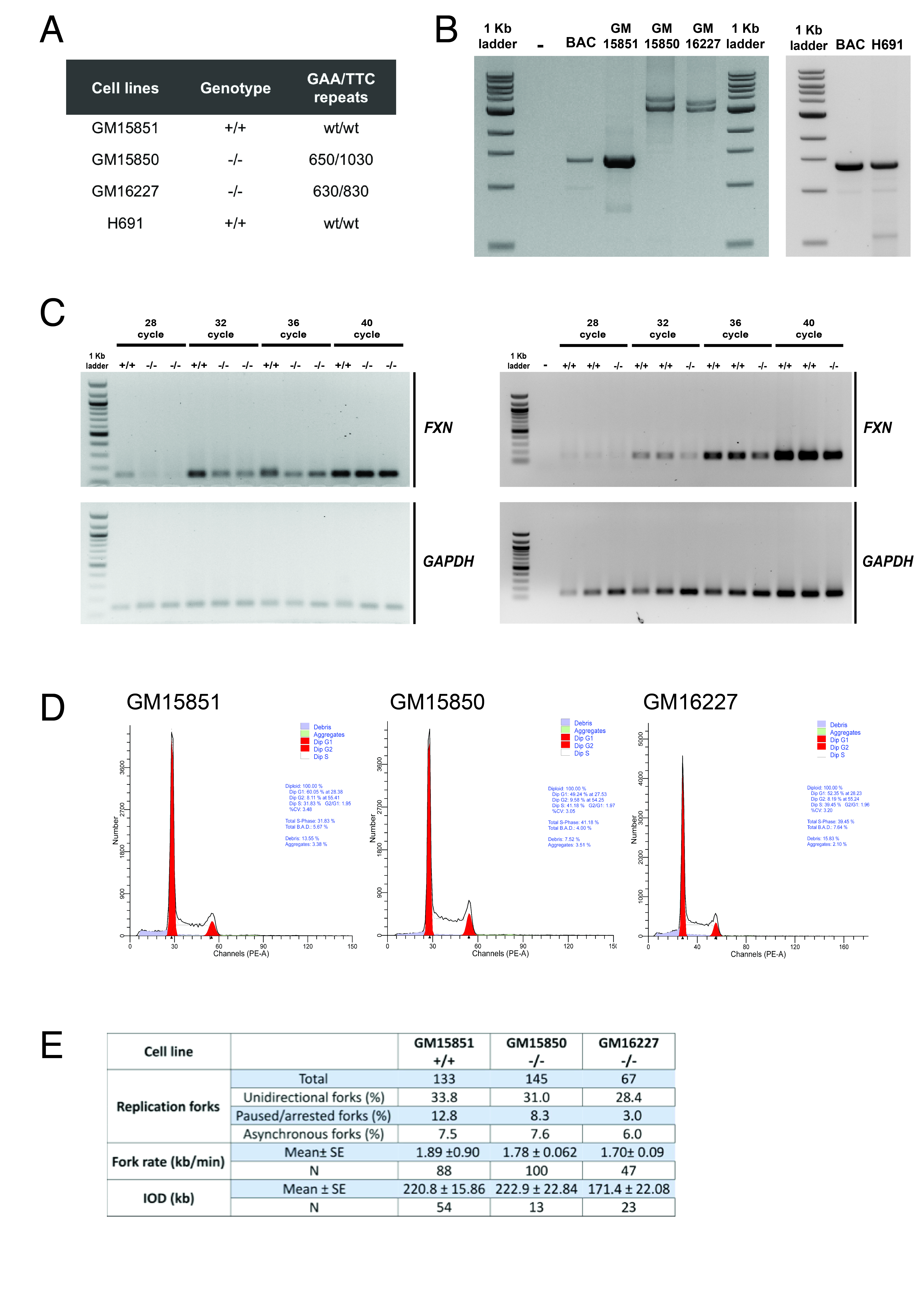

Supplement: S1 Fig — (A) The cell lines used in the study; information is provided by the Coriell repository apart from H691 cells; (B) the length of FXN alleles in the four cell lines, as evaluated by long-range PCR analysis; (C) transcriptional activity of FXN in the four cell lines, as evaluated by semiquantitative RT-PCR; (D) flow cytometry-based cell cycle profiles of the cell lines obtained from the Coriell repository; (E) genome-wide molecular combing analysis in the three cell lines obtained from the Coriell repository. (JPG) [file pgen.1006201.s001.jpg]

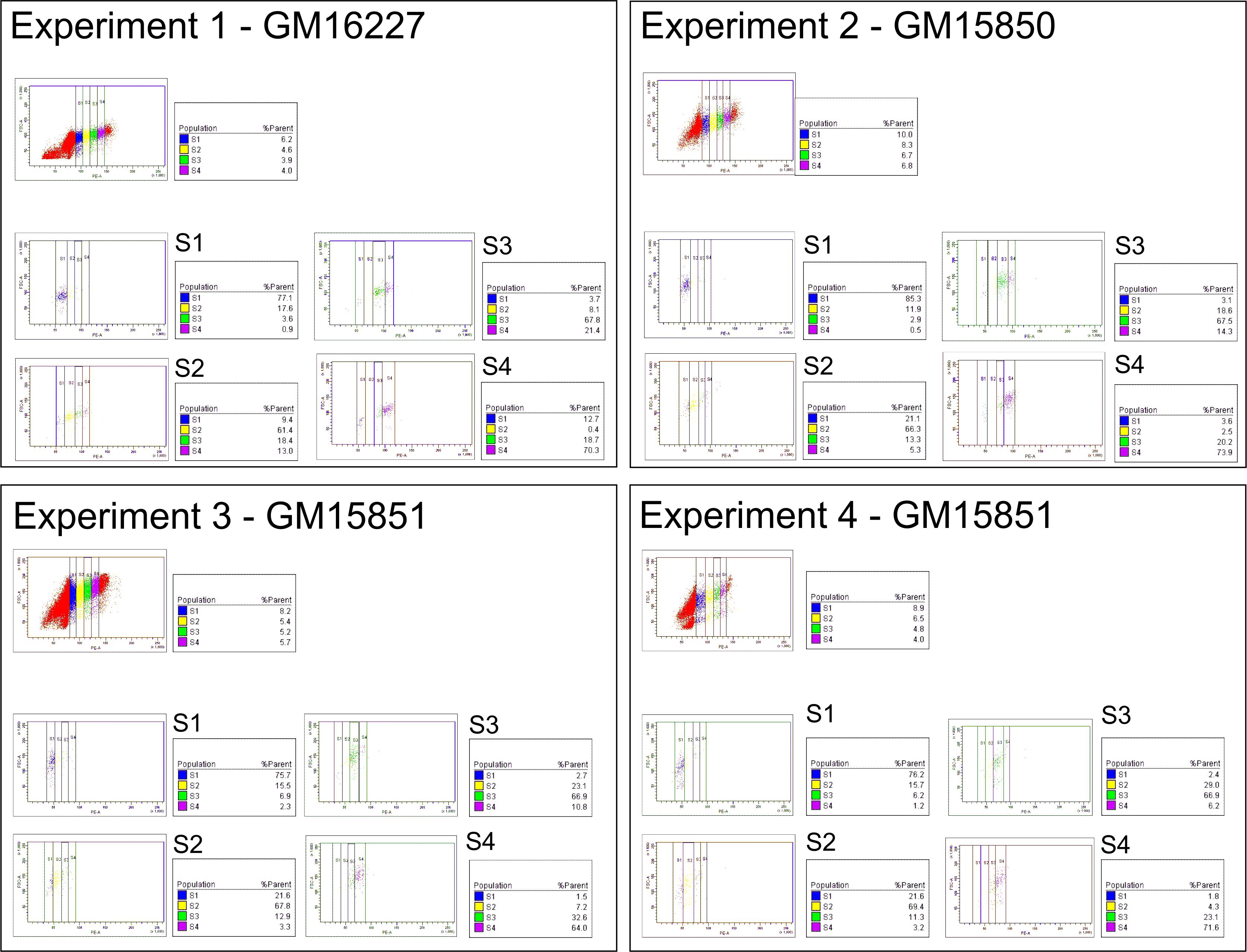

Supplement: S2 Fig — FACS sorting experiments aimed to separate cells in consecutive temporal windows of the S-phase (S1-S4). Experiments 3 and 4 represent two independent biological replicates in which GM15851 cells were separated by FACS sorting. (JPG) [file pgen.1006201.s002.jpg]

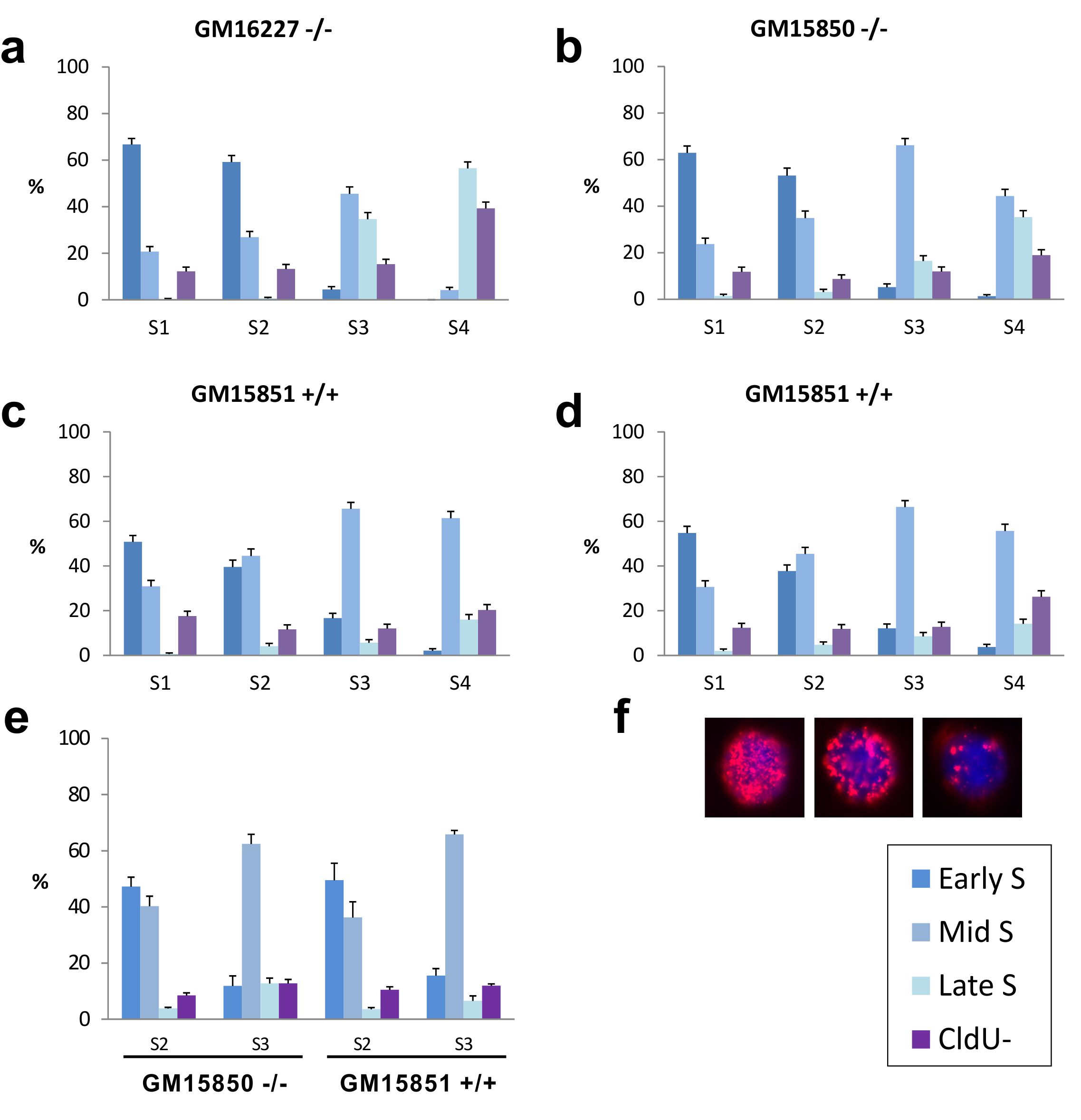

Supplement: S3 Fig — Before fractionation in four consecutive temporal windows of the S-phase (S5–S8, S2 Figs), cells were exposed to CldU for 30 min immediately before harvesting to confirm the accuracy of cell sorting procedure by CldU-immunodetection. Cells were classified in early, mid, late S-phase according to the observed fluorescent pattern (see F). (A-D) Quality control: data in the graphs were collected during the FISH analyses of FXN replication timing (respectively: experiments 1–4 summarized in S2 Fig; raw data in Supplementary Table 2). In all of the experiments each cell fraction is enriched for the expected S-phase stage (error bars = errors of percentages). (E) Reproducibility: average proportions of cells belonging to different stages of the S-phase, as observed in the course of three FISH experiments carried out independently on the same S2 and S3 cell samples (respectively: exp. 2 for GM15850 cells, exp. 3 for GM15851). (F) Examples (left to right) of early, mid, late S-phase nuclei according to the observed fluorescent CldU pattern. (JPG) [file pgen.1006201.s003.jpg]

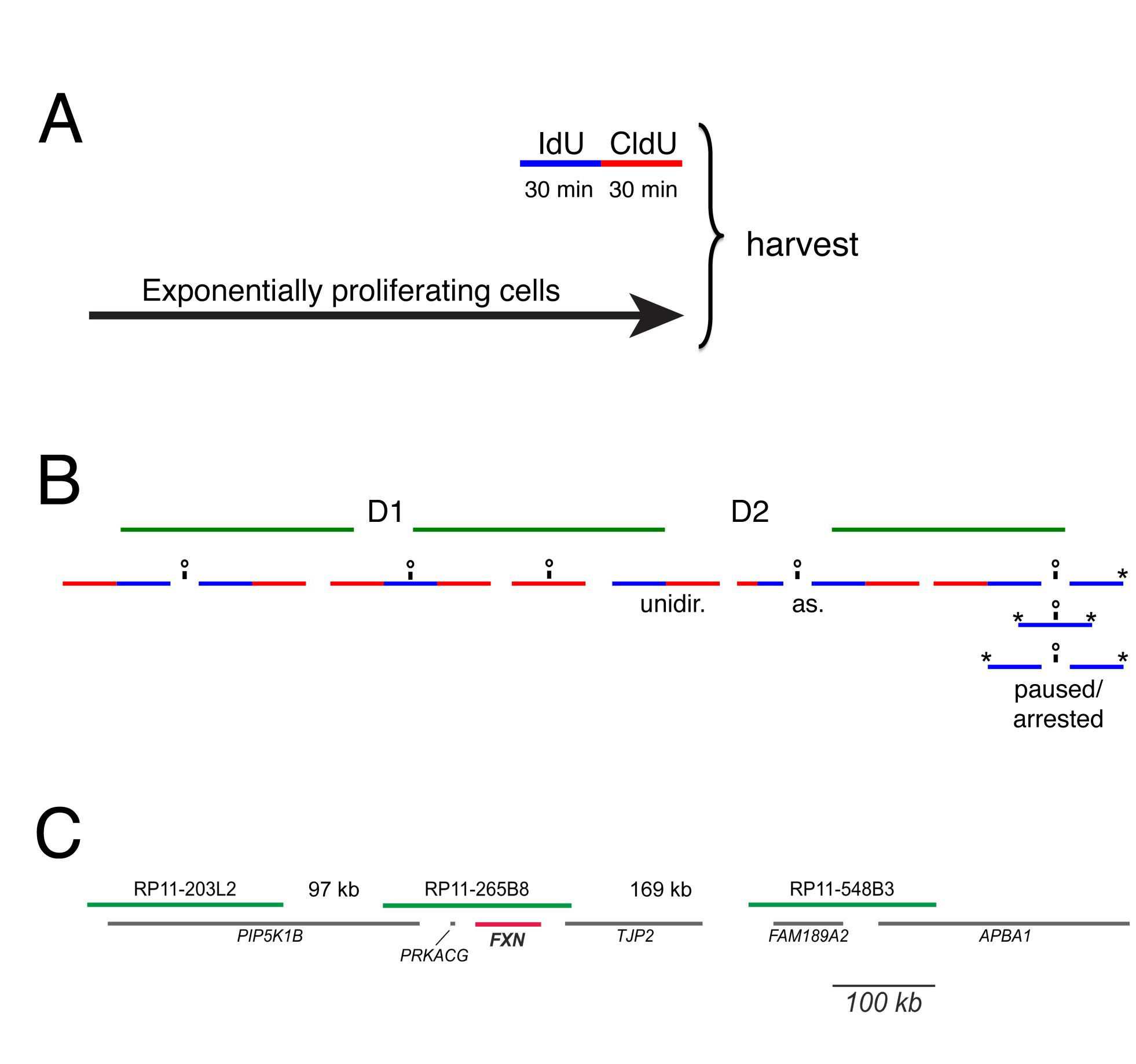

Supplement: S4 Fig — (A) The two-pulse labeling scheme for detection of replication forks. In the first pulse IdU is incorporated in the nascent strands and labeled DNA is detected by blue fluorescence; during the second pulse CldU is available for the synthesis of DNA, and labeling is detected by red fluorescence. (B) Examples of normal and altered replication patterns expected in single-locus replication analyses; replication tracks and probes are represented slightly displaced for simplicity. Three probes differentially spaced (D1 and D2) are detected by green fluorescence: the FISH pattern allows us to define the centromere-telomere orientation and the integrity of the molecule (a Cy5-labeled central probe is cohybridized with the biotin-labeled probes, to allow the centromere-telomere orientation when only two hybridization signals can be visualized). Bidirectional origins (o) may be mapped in the middle of the two arms or in the middle of a blue track of a replication fork. Paused/arrested forks (*) may be unilateral or bilateral events, as illustrated. Asynchronous forks (as.) fire from the origin with different rates. Unidirectional forks (unidir.) are identified when a single arm with blue/red pattern is progressing with same orientation than the upstream or downstream track (which is the case represented in this example). (C) The genomic region investigated in this study. Green lines represent three probes covering about 850 kb in the bp interval 68643,187–69477,097 at 9q21. Frataxin (FXN) is shown in red and the other genes mapping in the flanking regions are shown in grey. (JPG) [file pgen.1006201.s004.jpg]

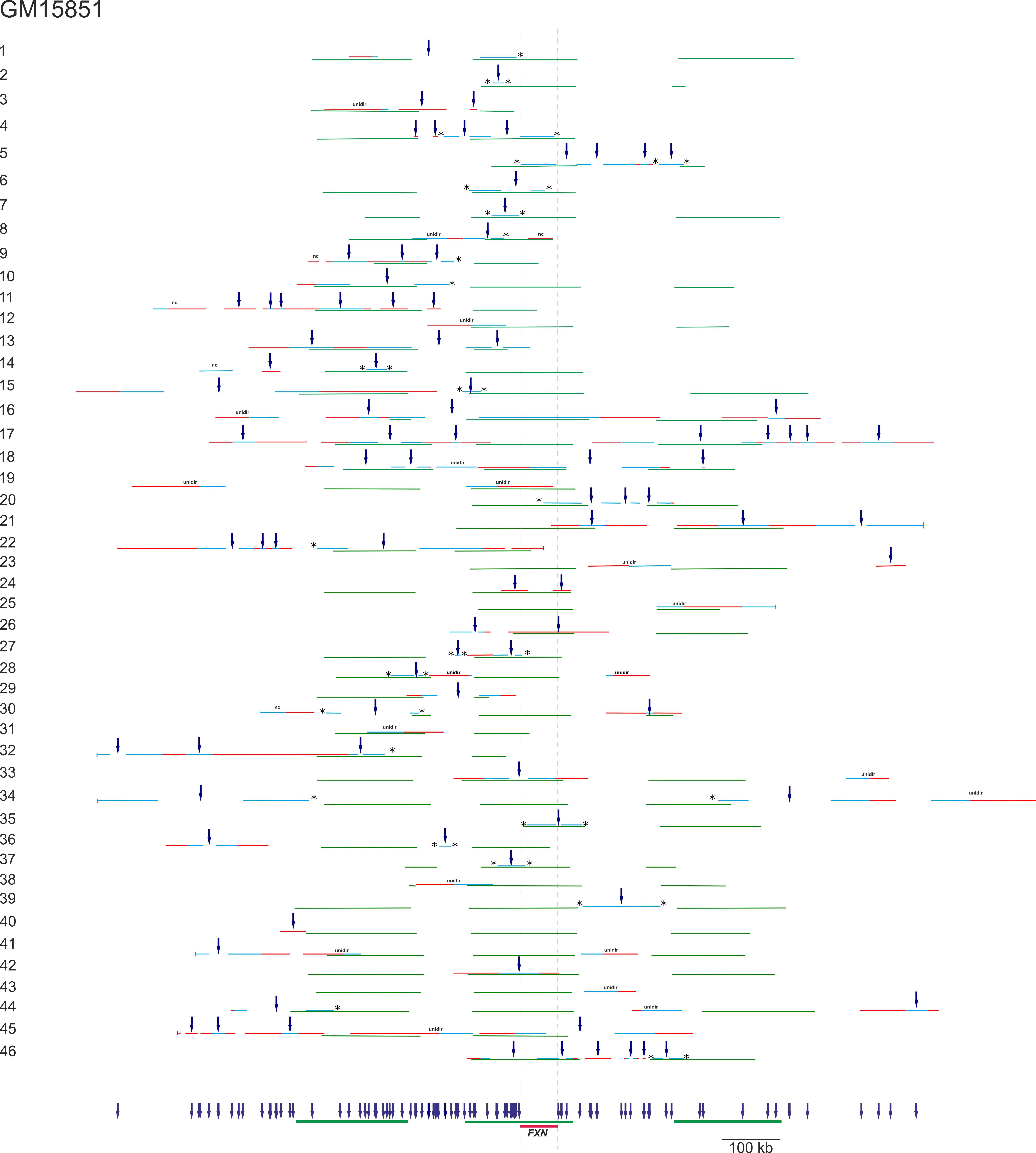

Supplement: S5 Fig — Dotted lines indicate the boundaries of the FXN sequence. A scheme of the genomic region is shown in S4C Fig Original reconstructed images are deposited in the Dryad Digital Repository at http://dx.doi.org/10.5061/dryad.f12cg. (JPG) [file pgen.1006201.s005.jpg]

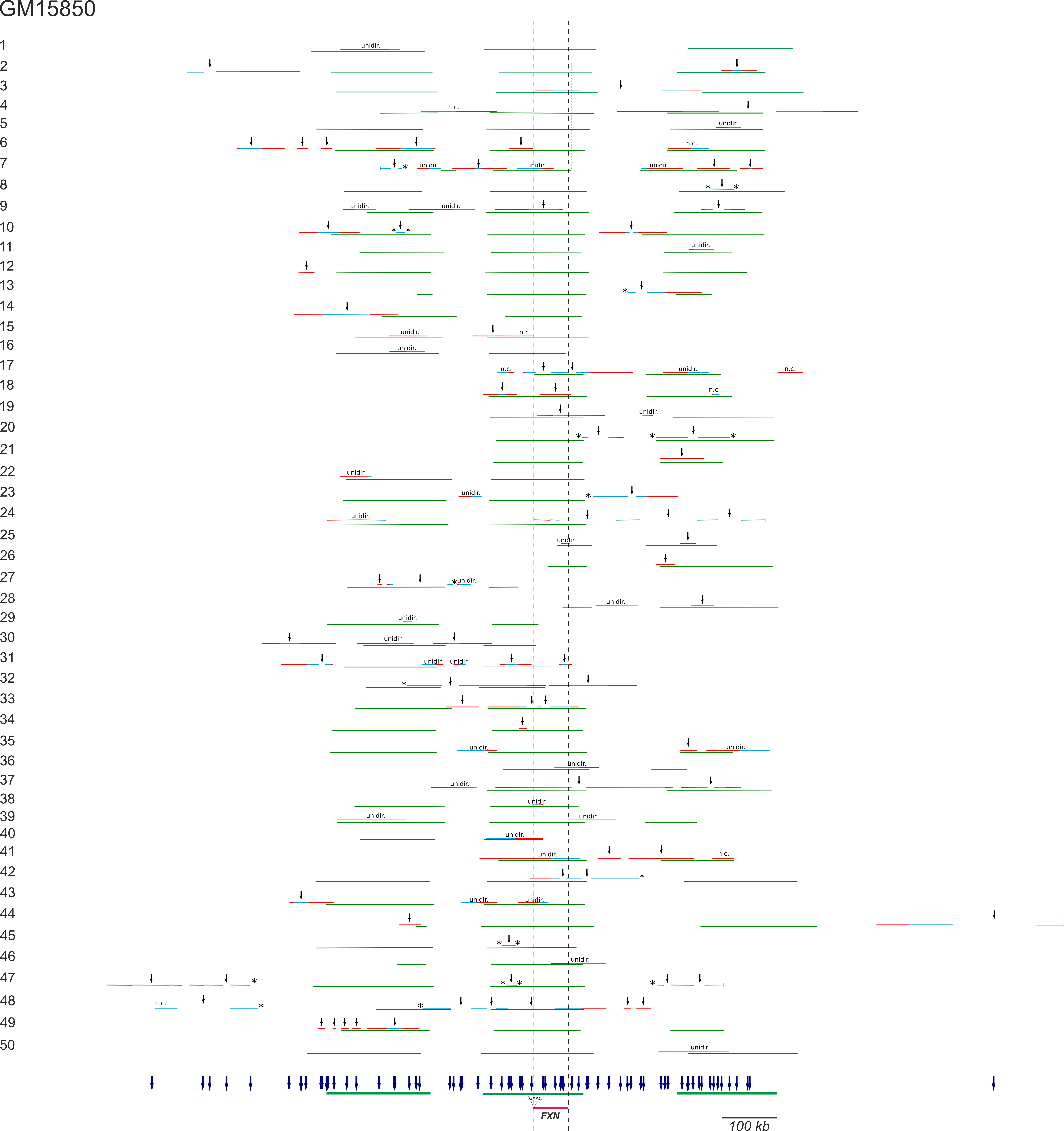

Supplement: S7 Fig — Dotted lines indicate the boundaries of the FXN sequence. A scheme of the genomic region is shown in S4C Fig Original reconstructed images are deposited in the Dryad Digital Repository at http://dx.doi.org/10.5061/dryad.f12cg. (JPG) [file pgen.1006201.s007.jpg]

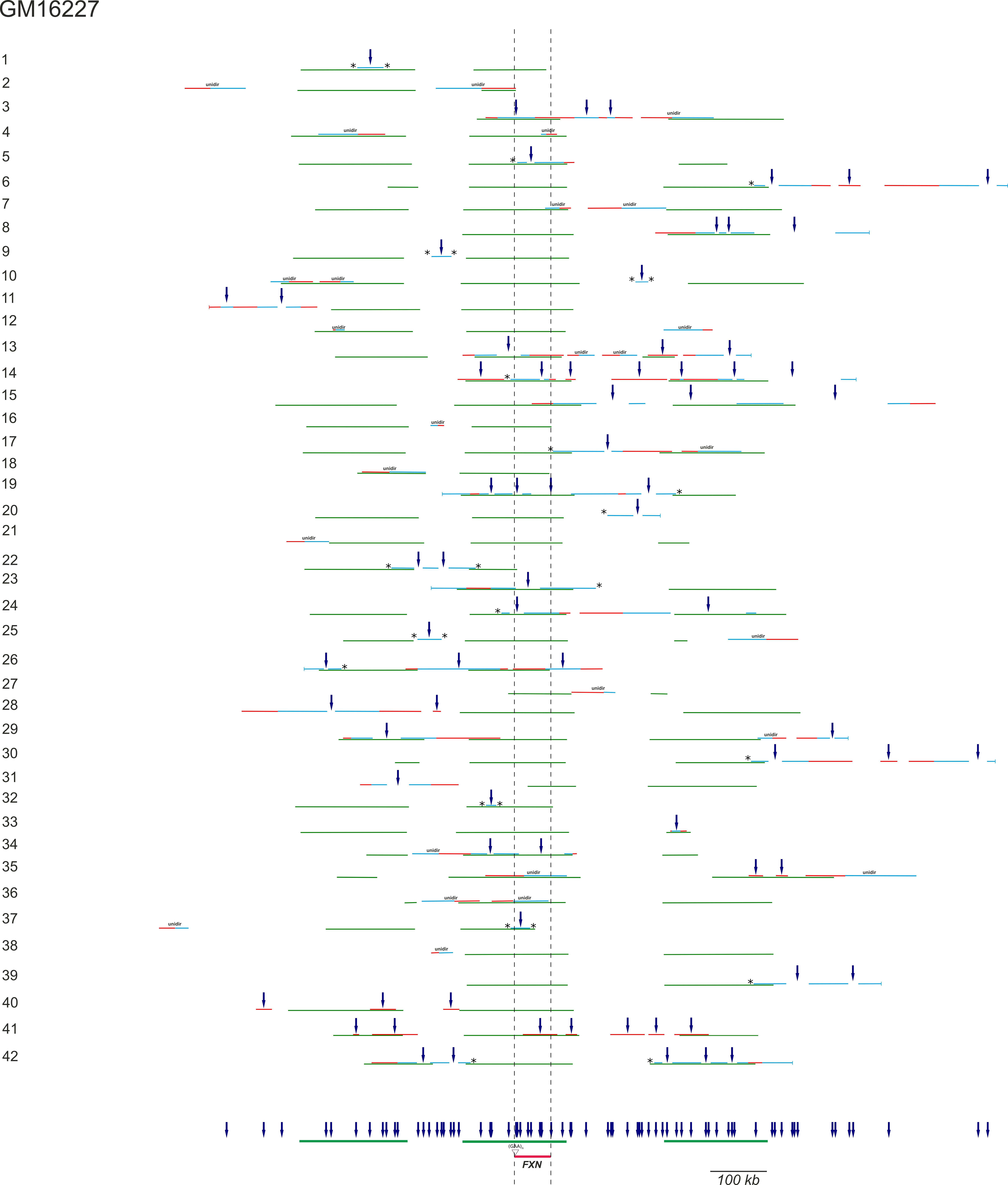

Supplement: S8 Fig — Dotted lines indicate the boundaries of the FXN sequence. A scheme of the genomic region is shown in S4C Fig Original reconstructed images are deposited in the Dryad Digital Repository at http://dx.doi.org/10.5061/dryad.f12cg. (JPG) [file pgen.1006201.s008.jpg]

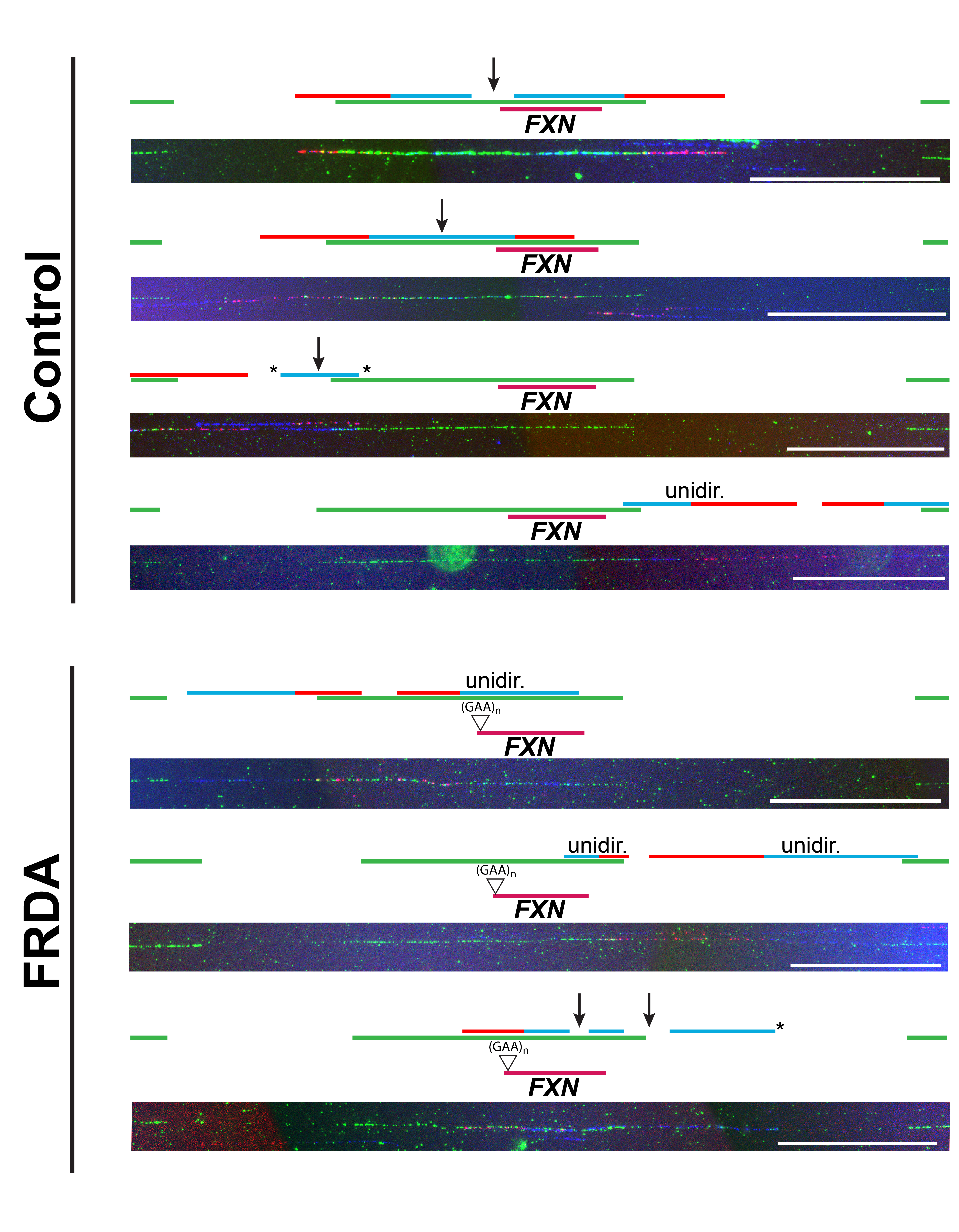

Supplement: S9 Fig — Images are selected enlargements from complete reconstructed molecules (from top to bottom: molecules 33, 42, 15 in S5 Fig, molecule 13 in S6 Fig, molecules 36 and 7 in S8 Fig, molecule 42 in S7 Fig) The probe (green), containing the FXN gene (red), and the flanking regions (probe-to-probe distances, S4C Fig) are shown. Replication tracks are visualized in blue (IdU) and red (CldU), arrows indicate origin positions, the asterisk corresponds to a paused/arrested fork, unidirectional forks are indicated. GAA-repeat expansion is also displayed. Calibration bar = 100kb. (JPG) [file pgen.1006201.s009.jpg]

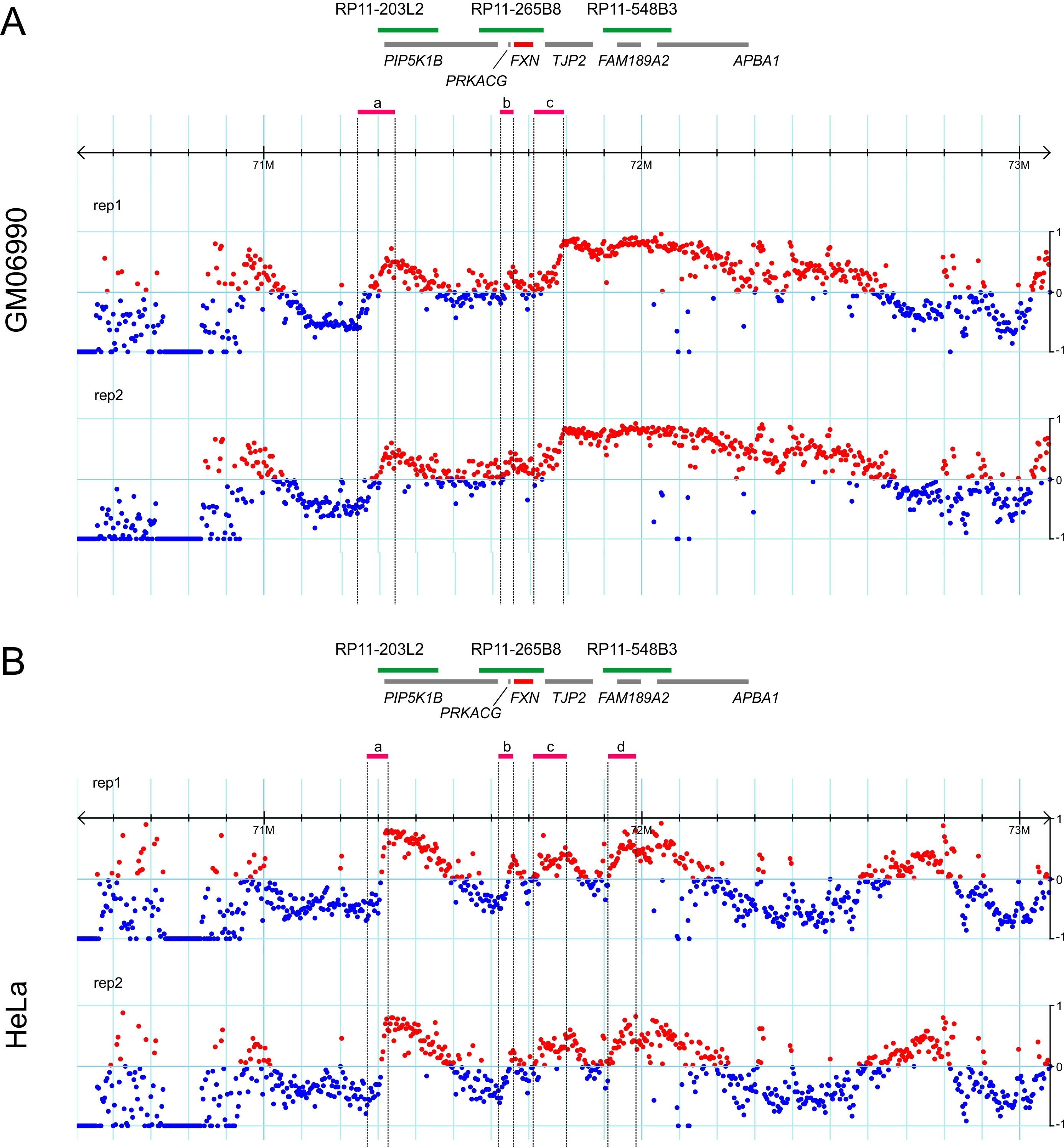

Supplement: S10 Fig — (A) RFD profile of the GM06990 lymphoblastoid cell line. Green lines represent the three BAC clones used as probes in molecular combing experiments, gray lines display genes, FXN is highlighted in red. Pink bars (a, b, c) represent initiation zones derived by OK-Seq analysis [6]. Two replicas are shown. (B) RFD profile of HeLa cell line. Green lines represent the three BAC clones used as probes in molecular combing experiments, gray lines display genes, FXN is highlighted in red. Pink bars (a, b, c, d) represent initiation zones derived by OK-Seq analysis [6]. Two replicas are shown. RFD were downloaded from http://157.136.54.88/cgi-bin/gbrowse/gbrowse/okazaki_ref/. (JPG) [file pgen.1006201.s010.jpg]
