# Supplementary material for: The Replication of Frataxin Gene Is Assured by Activation of Dormant Origins in the Presence of a GAA-Repeat Expansion
Source: PLoS Genet. 2016 Jul 22;12(7):e1006201. doi: 10.1371/journal.pgen.1006201 (PMC4957762; doi:10.1371/journal.pgen.1006201)
Supplement: S1 Table — (DOCX) [file pgen.1006201.s011.docx]

**S1 Table. Raw data of the replication timing analysis of *FXN* carried out by interphase FISH.**

| **Locus** | **Cell line,  *FXN* genotype** | **Total cells** | **Cells with SS pattern**  **N (% ± SE)** | **Cells with SD pattern**  **N (% ± SE)** | **Cells with DD pattern**  **N (% ± SE)** | **Cells with other patterns**  **N (% ± SE)** |
| --- | --- | --- | --- | --- | --- | --- |
| *FXN* | GM15851, control**^§^** | 288 | 65  (22.6 ± 2.46) | 110  (38.2 ± 2.86) | 97  (33.7 ± 2.78) | 16  5.6 ± 1.35 |
|  | GM15851, control**^ç^** | 273 | 78  (28.6 ± 2.73) | 98  (35.9 ± 2.90) | 84  (30.8 ± 2.79) | 13  (4.8 ± 1.29) |
|  | GM16227, FRDA | 252 | 76  (30.2 ± 2.89) | 81  (32.1 ± 2.94) | 36  (14.3 ± 2.20) | 59  (23.4 ± 2.67) |
|  | GM15850, FRDA**°** | 247 | 74  (30.0 ± 2.91) | 80  (32.4 ± 2.98) | 82  (33.2 ± 3.00) | 11  (4.5 ±1.31) |
|  | GM15850, FRDA | 373 | 94  (25.2 ± 2.25) | 136  (36.5 ± 2.49) | 117  (31.4 ± 2.40) | 26  (7.0 ± 1.32) |
| *FRA3B^#^* | GM15851, control**^§^** | 294 | 97  (33.0 ± 2.74) | 137  (46.6 ± 2.90) | 46  (15.6 ± 2.12) | 14  (4.8 ± 1.24) |
|  | GM15851, control **^ç^** | 288 | 86  (29.9 ± 2.70) | 120  (41.7± 2.91) | 71  (24.7 ± 2.54) | 11  (3.8 ± 1.13) |

^§^ Pre-sorting control population of Experiment 3 (see Supplementary Figure 2)

^ç^ Pre-sorting control population of Experiment 4 (see Supplementary Figure 2)

° Pre-sorting control population of Experiment 1 (see Supplementary Figure 2)

*^#^* The late replicating common fragile site *FRA3B* has been evaluated as positive control
