# Supplementary material for: The Replication of Frataxin Gene Is Assured by Activation of Dormant Origins in the Presence of a GAA-Repeat Expansion
Source: PLoS Genet. 2016 Jul 22;12(7):e1006201. doi: 10.1371/journal.pgen.1006201 (PMC4957762; doi:10.1371/journal.pgen.1006201)
Supplement: S2 Table — (DOCX) [file pgen.1006201.s012.docx]

**S2 Table. Raw data of the replication timing analysis of *FXN* carried out by interphase FISH after FACS cell sorting.**

| **Sorting experiment^ç^** | **Cell fraction** | **Replication patterns^#^** | **Total cells** | **S-phase cells^#^**  **N** | **early S-phase^#^**  **N**  **(% ± SE)^§^** | **mid S-phase^#^**  **N**  **(% ± SE)^§^** | **late S-phase^#^**  **N**  **(% ± SE)^§^** | **Hybridization efficiency** |
| --- | --- | --- | --- | --- | --- | --- | --- | --- |
| Experiment 1  (GM16227, FRDA cells) | S1 | SS | 175 | 154 | 146  (63.8 ± 3.18) | 8  (11.3 ± 3.75) | 0 | 91.1 |
|  |  | DD | 10 | 6 | 0 | 6  (8.5 ± 3.30) | 0 |  |
|  |  | SD | 108 | 100 | 58  (25.3 ± 2.87) | 41  (57.7 ± 5.86) | 1 |  |
|  |  | Others | 50 | 41 | 25  (10.9 ± 2.06) | 16  (22.5 ± 4.96) | 0 |  |
|  |  | Total | 343 | 301 | 229 | 71 | 1 |  |
|  | S2 | SS | 109 | 89 | 82  (43.9 ± 3.63) | 7  (8.2 ± 2.98) | 0 | 89.9 |
|  |  | DD | 21 | 15 | 2  (1.1 ± 0.75) | 12  (14.1 ± 3.78) | 1 |  |
|  |  | SD | 137 | 130 | 76  (40.6 ± 3.59) | 53  (62.4 ± 5.26) | 1 |  |
|  |  | Others | 49 | 40 | 27  (14.4 ± 2.57) | 13  (15.3 ± 3.90) | 0 |  |
|  |  | Total | 316 | 274 | 187 | 85 | 2 |  |
|  | S3 | SS | 21 | 16 | 9  (69.2 ± 12.80) | 7  (5 ± 1.92) | 0 | 93.0 |
|  |  | DD | 115 | 95 | 0 | 32  (23.9 ± 3.68) | 63  (61.8 ± 4.81) |  |
|  |  | SD | 120 | 108 | 1  (7.7 ± 7.39) | 79  (59.0 ± 4.25) | 28  (27.5 ± 4.42) |  |
|  |  | Others | 38 | 30 | 3  (23.1 ±11.68) | 16  (11.9 ± 2.80) | 11  (10.8 ± 3.07) |  |
|  |  | Total | 294 | 249 | 13 | 134 | 102 |  |
|  | S4 | SS | 1 | 0 | 0 | 0 | 0 | 94.6 |
|  |  | DD | 247 | 155 | 0 | 1  (7.1 ± 6.88) | 154  (82.4 ± 2.79) |  |
|  |  | SD | 55 | 29 | 0 | 8  (57.1 ± 13.23) | 21  (11.2 ± 2.31) |  |
|  |  | Others | 28 | 17 | 0 | 5  (35.7 ± 12.81) | 12  (6.4 ± 1.79) |  |
|  |  | Total | 331 | 201 | 0 | 14 | 187 |  |
| Experiment 2  (GM15850, FRDA cells) | S1 | SS | 136 | 116 | 105  (60.0 ± 3.70) | 11  (17 ± 4.59) | 0 | 94.8 |
|  |  | DD | 11 | 7 | 0 | 4  (6 ± 2.94) | 3 |  |
|  |  | SD | 104 | 101 | 61  (34.9 ± 3.60) | 39  (59.1 ± 6.05) | 1 |  |
|  |  | Others | 27 | 21 | 9  (5.1 ± 1.67) | 12  (18.2 ± 4.75) | 0 |  |
|  |  | Total | 278 | 245 | 175 | 66 | 4 |  |
|  | S2 | SS | 105 | 104 | 96  (71.6 ± 3.89) | 8  (9.1 ± 3.06) | 0 | 96.0 |
|  |  | DD | 28 | 19 | 0 | 14  (15.9 ± 3.90) | 5 |  |
|  |  | SD | 101 | 91 | 26  (19.4 ± 3.42) | 63  (71.6 ± 4.81) | 2 |  |
|  |  | Others | 18 | 16 | 12  (9.0 ± 2.47) | 3  (3.4 ± 1.93) | 1 |  |
|  |  | Total | 252 | 230 | 134 | 88 | 8 |  |
|  | S3 | SS | 12 | 12 | 10  (67.0 ± 12.17) | 2  (1.1 ± 0.75) | 0 | 97.9 |
|  |  | DD | 124 | 109 | 1  (7.0 ± 6.44) | 68  (36.2 ± 3.50) | 40  (85 ± 5.19) |  |
|  |  | SD | 137 | 123 | 4  (26.7 ± 11.42) | 113  (60.1 ± 3.57) | 6  (12.8 ± 4.87) |  |
|  |  | Others | 11 | 6 | 0 | 5  (2.7 ± 1.17) | 1  (2.1 ±2.10) |  |
|  |  | Total | 284 | 250 | 15 | 188 | 47 |  |
|  | S4 | SS | 8 | 4 | 3 | 1  (0.8 ± 0.75) | 0 | 97.7 |
|  |  | DD | 189 | 154 | 0 | 64  (48.1 ± 4.33) | 90  (85.0 ± 3.48) |  |
|  |  | SD | 89 | 73 | 1 | 62  (46.6 ± 4.33) | 10  (9.4 ± 2.84) |  |
|  |  | Others | 14 | 12 | 0 | 6  (4.5 ± 1.80) | 6  (5.7 ± 2.24) |  |
|  |  | Total | 300 | 243 | 4 | 133 | 106 |  |
| Experiment 3  (GM15851, controls) | S1 | SS | 121 | 96 | 90  (57.7 ± 3.96) | 6  (6.3 ± 2.50) | 0 | 96.1 |
|  |  | DD | 21 | 17 | 0 | 15  (15.8 ± 3.74) | 2 |  |
|  |  | SD | 142 | 121 | 56  (35.9 ± 3.84) | 65  (68.4 ± 4.77) | 0 |  |
|  |  | Others | 23 | 19 | 10  (6.4 ± 1.96) | 9  (9.5 ± 3.00) | 0 |  |
|  |  | Total | 307 | 253 | 156 | 95 | 2 |  |
|  | S2 | SS | 67 | 57 | 50  (47.6 ± 4.87) | 7  (5.9 ± 2.17) | 0 | 97.5 |
|  |  | DD | 51 | 39 | 1   1. ± 0.95) | 29  (24.6 ± 3.96) | 9  (81.8 ± 11.63) |  |
|  |  | SD | 136 | 128 | 51  (48.6 ± 4.88) | 75  (63.6 ± 4.43) | 2  (18.2 ± 11.63) |  |
|  |  | Others | 11 | 10 | 3  (2.9 ± 1.63) | 7  (5.9 ± 2.17) | 0 |  |
|  |  | Total | 265 | 234 | 105 | 118 | 11 |  |
|  | S3 | SS | 24 | 15 | 9  (19.1 ± 5.74) | 6  (3.2 ± 1.30) | 0 | 97.0 |
|  |  | DD | 95 | 83 | 1  (2.1 ± 2.10) | 70  (37.8 ± 3.57) | 12  (75.0 ± 10.83) |  |
|  |  | SD | 146 | 136 | 33  (70.2± 6.67) | 99±  (53.5 ± 3.67) | 4  (25.0 ± 10.83) |  |
|  |  | Others | 17 | 14 | 4  (8.5 ± 4.07) | 10  (5.4 ± 1.66) | 0 |  |
|  |  | Total | 282 | 248 | 47 | 185 | 16 |  |
|  | S4 | SS | 9 | 8 | 2 | 6  (3.5 ± 1.40) | 0 | 97.5 |
|  |  | DD | 176 | 139 | 0 | 97  (56.4 ± 3.78) | 42  (93.3 ± 3.72) |  |
|  |  | SD | 84 | 68 | 4 | 62  (36.0 ± 3.66) | 2  (4.4 ± 3.07) |  |
|  |  | Others | 11 | 8 | 0 | 7  (4.1 ± 1.51) | 1  (2.2 ± 2.20) |  |
|  |  | Total | 280 | 223 | 6 | 172 | 45 |  |
| Experiment 4  (GM15851, controls) | S1 | SS | 123 | 105 | 103  (64.8 ± 3.78) | 2  (2.2 ± 1.57) | 0 | 98.4 |
|  |  | DD | 23 | 15 | 0 | 9  (10.1 ± 3.20) | 6 |  |
|  |  | SD | 136 | 128 | 55  (34.6 ± 3.77) | 73  (82.0± 4.07) | 0 |  |
|  |  | Others | 8 | 6 | 1  (0.6 ± 0.63) | 5  (5.6 ± 2.44) | 0 |  |
|  |  | Total | 290 | 254 | 159 | 89 | 6 |  |
|  | S2 | SS | 74 | 58 | 55  (47.0 ± 4.61) | 3  (2.1 ± 1.22) | 0 | 97.3 |
|  |  | DD | 57 | 51 | 1  (0.9 ± 0.85) | 38  (27.0 ± 3.74) | 12  (80.0 ± 10.33) |  |
|  |  | SD | 164 | 151 | 57  (48.7 ± 4.62) | 91  (64.5 ± 4.03) | 3  (20.0 ± 10.33) |  |
|  |  | Others | 15 | 13 | 4  (3.4 ± 1.68) | 9  (6.4 ± 2.06) | 0 |  |
|  |  | Total | 310 | 273 | 117 | 141 | 15 |  |
|  | S3 | SS | 24 | 15 | 11  (32.4 ± 8.02) | 4  (2.2 ± 1.06) | 0 | 98.8 |
|  |  | DD | 114 | 96 | 0 | 72  (38.7 ± 3.57) | 24 |  |
|  |  | SD | 135 | 128 | 22  (64.7 ± 8.20) | 106  (57.0 ± 3.63) | 0 |  |
|  |  | Others | 7 | 5 | 1  (2.9 ± 2.90) | 4  (2.2 ± 1.06) | 0 |  |
|  |  | Total | 280 | 244 | 34 | 186 | 24 |  |
|  | S4 | SS | 13 | 3 | 2  (18.2 ± 11.63) | 1  (0.6 ± 0.62) | 0 | 98.8 |
|  |  | DD | 170 | 124 | 1  (9.1 ± 8.67) | 85  (52.8 ± 3.93) | 38  (92.7 ± 4.07) |  |
|  |  | SD | 99 | 81 | 7  (63.6 ± 14.50) | 71  (44.1 ± 3.91) | 3  (7.3 ± 4.07) |  |
|  |  | Others | 7 | 5 | 1  (9.1 ±8.67) | 4  (2.5 ± 1.23) | 0 |  |
|  |  | Total | 289 | 213 | 11 | 161 | 41 |  |

^ç^ As described in Supplementary Figure 2

# Replication patterns are based on features of the FISH signals; S-phase cells are classified according to CldU-labelling. All details in Materials and Methods

^§^ Percentages and SE of percentages were calculated only if > 10 total cells were observed per each S-phase substage
