# Supplementary material for: The Replication of Frataxin Gene Is Assured by Activation of Dormant Origins in the Presence of a GAA-Repeat Expansion
Source: PLoS Genet. 2016 Jul 22;12(7):e1006201. doi: 10.1371/journal.pgen.1006201 (PMC4957762; doi:10.1371/journal.pgen.1006201)
Supplement: S3 Table — (DOCX) [file pgen.1006201.s013.docx]

**S3 Table. Replication timing of the late replicating sequence *FRA3B* according to interphase FISH after FACS cell sorting.**

| **Cell sample** | **Cell fraction** | **Replication patterns^#^** | **Total cells** | **S-phase cells^#^**  **N** | **early S-phase^#^**  **N**  **(% ± SE)^§^** | **mid S-phase^#^**  **N**  **(% ± SE)^§^** | **late S-phase^#^**  **N**  **(% ± SE)^§^** |
| --- | --- | --- | --- | --- | --- | --- | --- |
| Control  (GM15851 cells) | S1 | SS | 131 | 97 | 90  (61.2 ± 4.02) | 7  (9.9 ±3.54) | 0 |
|  |  | DD | 20 | 16 | 1  (0.7 ± 0.68) | 10  (14.1 ±4.1) | 5 |
|  |  | SD | 125 | 102 | 49  (33.3 ± 3.89) | 53  (74.6 ±5.16) | 0 |
|  |  | Others | 10 | 8 | 7  (4.8 ± 1.76) | 1  (1.4 ±1.40) | 0 |
|  |  | Total | 286 | 223 | 147 | 71 | 5 |
|  | S2 | SS | 101 | 94 | 86  (61.9 ± 4.12) | 8  (7.3 ± 2.48) | 0 |
|  |  | DD | 27 | 24 | 1  (0.7 ± 0.72) | 15  (13.6 ± 3.27) | 8  (66.7 ± 13.61) |
|  |  | SD | 145 | 132 | 49  (35.3 ± 4.05) | 80  (72.7 ± 4.25) | 3  (25.0 ± 12.50) |
|  |  | Others | 12 | 11 | 3  (2.2 ± 1.23) | 7  (6.4 ± 2.33) | 1  (8.3 ± 7.98) |
|  |  | Total | 285 | 261 | 139 | 110 | 12 |
|  | S3 | SS | 39 | 27 | 21  (37.5 ± 6.47) | 6  (3.2 ± 1.30) | 0 |
|  |  | DD | 85 | 68 | 1  (1.8 ± 1.78) | 57  (30.6 ± 3.38) | 10  (83.3 ± 10.76) |
|  |  | SD | 156 | 147 | 31  (55.4 ± 6.64) | 115  (61.8 ± 3.56) | 1  (8.3 ± 7.98) |
|  |  | Others | 12 | 12 | 3  (5.4 ± 3.01) | 8  (4.3 ± 1.49) | 1  (8.3 ± 7.98) |
|  |  | Total | 292 | 254 | 56 | 186 | 12 |
|  | S4 | SS | 11 | 3 | 1  (8.3 ± 7.98) | 2   - 1. ± 0.76) | 0 |
|  |  | DD | 164 | 127 | 0 | 85  (46.2 ± 3.68) | 42  (84.0 ± 5.19) |
|  |  | SD | 130 | 110 | 11  (91.7 ± 7.98) | 92  (50.0 ± 3.69) | 7  (14.0 ± 4.91) |
|  |  | Others | 10 | 6 | 0 | 5  (2.7 ± 1.20) | 1  (2.0 ± 1.98) |
|  |  | Total | 315 | 246 | 12 | 184 | 50 |
| FRDA  (15850 cells) | S1 | SS | 144 | 116 | 111  (77.1 ± 3.50) | 5  (6.8 ± 2.96) | 0 |
|  |  | DD | 7 | 2 | 0 | 0 | 2 |
|  |  | SD | 94 | 88 | 28  (19.4 ± 3.30) | 60  (82.2 ± 4.48) | 0 |
|  |  | Others | 16 | 13 | 5  (3.5 ± 1.53) | 8  (11.0 ± 3.66) | 0 |
|  |  | Total | 261 | 219 | 144 | 73 | 2 |
|  | S2 | SS | 84 | 74 | 66  (64.7 ± 4.73) | 8  (7.0 ± 2.37) | 0 |
|  |  | DD | 18 | 17 | 0 | 11  (9.6 ± 2.74) | 6  (54.5 ± 15.01) |
|  |  | SD | 125 | 121 | 28  (27.5 ± 4.42) | 89  (77.4 ± 3.90) | 4  (36.4 ± 14.50) |
|  |  | Others | 18 | 16 | 8  (7.8 ± 2.66) | 7  (6.1 ± 2.23) | 1  (9.1 ± 8.67) |
|  |  | Total | 245 | 228 | 102 | 115 | 11 |
|  | S3 | SS | 26 | 18 | 16  (48.5 ± 8.70) | 2  (1.2 ± 0.86) | 0 |
|  |  | DD | 73 | 60 | 0 | 38  (23.3 ± 3.31) | 22  (84.6 ± 7.08) |
|  |  | SD | 134 | 130 | 16  (48.5 ± 8.70) | 111  (68.1 ± 3.65) | 3  (11.5 ± 6.27) |
|  |  | Others | 16 | 14 | 1  (3.0 ± 2.98) | 12  (7.4 ± 2.05) | 1  (3.8 ± 3.77) |
|  |  | Total | 249 | 222 | 33 | 163 | 26 |
|  | S4 | SS | 3 | 2 | 1 | 1  (0.7 ± 0.70) | 0 |
|  |  | DD | 138 | 108 | 0 | 47  (33.1 ± 3.95) | 61  (92.4 ± 3.26) |
|  |  | SD | 112 | 92 | 0 | 89  (62.7 ± 4.06) | 3  (4.5 ± 2.56) |
|  |  | Others | 11 | 8 | 1 | 5  (3.5 ± 1.55) | 2  (3.0 ± 2.11) |
|  |  | Total | 264 | 210 | 2 | 142 | 66 |

# Replication patterns are based on features of the FISH signals of BAC RP11-468L11; S-phase cells are classified according to CldU-labelling. All details in Materials and Methods

^§^ Percentages and SE of percentages were calculated only if > 10 total cells were observed per each S-phase substage
