# Supplementary material for: The Replication of Frataxin Gene Is Assured by Activation of Dormant Origins in the Presence of a GAA-Repeat Expansion
Source: PLoS Genet. 2016 Jul 22;12(7):e1006201. doi: 10.1371/journal.pgen.1006201 (PMC4957762; doi:10.1371/journal.pgen.1006201)
Supplement: S4 Table — (DOCX) [file pgen.1006201.s014.docx]

**S4 Table. Replication timing of a genomic domain located about 170 kb downstream the *FXN* allele, according to interphase FISH after FACS cell sorting.**

| **Cell sample** | **Cell fraction** | **Replication patterns^#^** | **Total cells** | **S-phase cells^#^**  **N** | **early S-phase^#^**  **N**  **(% ± SE)^§^** | **mid S-phase^#^**  **N**  **(% ± SE)^§^** | **late S-phase^#^**  **N**  **(% ± SE)^§^** |
| --- | --- | --- | --- | --- | --- | --- | --- |
| Control  (GM15851 cells) | S2 | SS | 114 | 95 | 95  (50.0 ± 3.67) | 0 | 0 |
|  |  | DD | 40 | 29 | 1  (0.5 ± 0.53) | 20  (24.7 ± 4.79) | 8 |
|  |  | SD | 152 | 146 | 90  (47.4 ± 3.62) | 56  (69.1 ± 5.13) | 0 |
|  |  | Others | 9 | 9 | 4  (2.1 ± 1.04) | 5  (6.2 ± 2.67) | 0 |
|  |  | Total | 315 | 279 | 190 | 81 | 8 |
|  | S3 | SS | 16 | 11 | 10  (29.4 ± 7.81) | 1  (0.5 ± 0.47) | 0 |
|  |  | DD | 134 | 111 | 0 | 82  (38.3 ± 3.32) | 29  (93.5 ± 4.41) |
|  |  | SD | 159 | 153 | 22  (64.7 ± 8.20) | 129  (60.3 ± 3.35) | 2  (6.5 ± 4.41) |
|  |  | Others | 4 | 4 | 2  (5.9 ± 4.04) | 2  (0.9 ± 0.66) | 0 |
|  |  | Total | 313 | 279 | 34 | 214 | 31 |
| FRDA  (15850 cells) | S2 | SS | 89 | 77 | 76  (53.5 ± 4.19) | 1  (0.8 ± 0.84) | 0 |
|  |  | DD | 48 | 39 | 2  (1.4 ± 0.99) | 26  (22.0 ± 3.82) | 11  (91.7 ± 7.98) |
|  |  | SD | 151 | 143 | 59  (41.5 ± 4.14) | 83  (70.3 ± 4.21) | 1  (8.3 ± 7.98) |
|  |  | Others | 14 | 13 | 5  (3.5 ± 1.55) | 8  (6.8 ± 2.31) | 0 |
|  |  | Total | 302 | 272 | 142 | 118 | 12 |
|  | S3 | SS | 27 | 14 | 13  (26.0 ± 6.20) | 1  (0.6 ± 0.62) | 0 |
|  |  | DD | 115 | 95 | 0 | 65  (40.1 ± 3.85) | 30  (90.9 ± 5.00) |
|  |  | SD | 135 | 127 | 35  (70.0 ± 6.48) | 90  (55.6 ± 3.90) | 2  (6.1 ± 4.15) |
|  |  | Others | 13 | 9 | 2  (4.0 ± 2.77) | 6  (3.7 ± 1.48) | 1  (3.0 ± 2.98) |
|  |  | Total | 290 | 245 | 50 | 162 | 33 |

# Replication patterns are based on features of the FISH signals of BAC RP11-548B3; S-phase cells are classified according to CldU-labelling. All details in Materials and Methods

^§^ Percentages and SE of percentages were calculated only if > 10 total cells were observed per each S-phase substage
