# Supplementary material for: The Replication of Frataxin Gene Is Assured by Activation of Dormant Origins in the Presence of a GAA-Repeat Expansion
Source: PLoS Genet. 2016 Jul 22;12(7):e1006201. doi: 10.1371/journal.pgen.1006201 (PMC4957762; doi:10.1371/journal.pgen.1006201)
Supplement: S5 Table — (DOCX) [file pgen.1006201.s015.docx]

|  |  | **Primer** | **Sequence** | **Position (GRCh38.p2)** | **Product (bp)** |
| --- | --- | --- | --- | --- | --- |
| **Chr.9**  **CTR** | upstream *FXN* | C1_for | 5'-AGGCAAGAGACAAGGCAAGACG-3' | 69027030 - 69027051 | 114 |
|  |  | C1_rev | 5'-TCTTCTCTGCCTCTGACCTGATG-3' | 69027121 - 69027143 |  |
|  | downstream *FXN* | C2_for | 5'-AGGTTGTAGTCACTGGTGGGTT-3' | 69205381 - 69205402 | 104 |
|  |  | C2_rev | 5'-ACAGCAGCCTTTGATCCACAGA-3' | 69205463 - 69205484 |  |
|  |  | C3_for | 5'-TGCAGACCATCACCAAGACAAA-3' | 69206988 - 69207010 | 65 |
|  |  | C3_rev | 5'-AGGAGGCAGAGTATGGTGGGAG-3' | 69207031 - 69207052 |  |
| **Chr.9**  ***FXN*** |  | F1_for | 5'-TCCTGTCACCACTTTCCTTCCA-3' | 69060619 - 69060640 | 91 |
|  |  | F1_rev | 5'-GCTCAACTTCCTCCCACCAGT-3' | 69060689 - 69060709 |  |
|  |  | F2_for | 5'-AACAAACCAGCAGTCCCAGATG-3' | 69064602 - 69064623 | 112 |
|  |  | F2_rev | 5'-GAGCTTTCCCTGTTCCCTGTGAG-3' | 69064691 - 69064713 |  |
|  |  | F3_for | 5'-AACCTGGGATTCTAGCAGCCTG-3' | 69086527 - 69086548 | 83 |
|  |  | F3_rev | 5'-AGGGATGAGGGGAACAGGGATC-3' | 69086588 - 69086609 |  |
|  |  | F4_for | 5'-ACTCTCCTGGCCTACTAGCTC-3' | 69087712 - 69087732 | 100 |
|  |  | F4_rev | 5'-GCTAGGTGATGTTATGAGGGGTCC-3' | 69087789 - 69087812 |  |
| **Chr.19**  ***LAMIN B2*** |  | LMNB2_for | 5'-GGCTGGCATGGACTTTCATTTCA-3' | 2428139 - 2428162 | 280 |
|  |  | LMNB2_rev | 5'-CTCAGGAATAAACTCAGAGGCAGA-3' | 2428288 - 2428310 |  |
|  |  | LB2C1_for ^§^ | 5'-GTTAACAGTCAGGCGCATGGG-3' | 2431926 - 2431947 | 240 |
|  |  | LB2C1_rev ^§^ | 5'-CCATCAGGGTCACCTCTGGTTC-3' | 2432142 - 2432166 |  |

**S5 Table. Oligonucleotide primer sequences.**

§ according to: Giacca et al. Proc Natl Acad Sci U S A. 1994 91:7119-7123.
